# K⊂{[Fe^II^(Tp)(CN)_3_]_4_[Co^III^(^pz^Tp)]_3_[Co^II^(^pz^Tp)]}: a neutral soluble model complex of photomagnetic Prussian blue analogues†
†This work has been conducted within a Co-tutelle framework between the Karlsruhe Institute of Technology (KIT), Karlsruhe, and Sorbonne Universités, UPMC – Paris 6, Paris.
‡
‡Electronic supplementary information (ESI) available: Additional details on NMR, EPR, X-ray diffraction, SQUID, and IR data. CCDC 1469358. For ESI and crystallographic data in CIF or other electronic format see DOI: 10.1039/c6sc01435f


**DOI:** 10.1039/c6sc01435f

**Published:** 2016-05-13

**Authors:** D. Garnier, J.-R. Jiménez, Y. Li, J. von Bardeleben, Y. Journaux, T. Augenstein, E. M. B. Moos, M. T. Gamer, F. Breher, R. Lescouëzec

**Affiliations:** a Institute of Inorganic Chemistry , Karlsruhe Institute of Technology (KIT) , Engesserstr. 15, Geb. 30.45 , D-76131 Karlsruhe , Germany . Email: breher@kit.edu; b Institut Parisien de Chimie Moléculaire – CNRS UMR 8232 , UPMC – Paris 6 , Sorbonne Universités , 4 place Jussieu , F-75252 Paris cedex 05 , France . Email: rodrigue.lescouezec@upmc.fr; c Institut des Nanosciences de Paris – CNRS UMR 7588 , UPMC – Paris 6 , Sorbonne Universités , 4 place Jussieu , F-75252 Paris cedex 05 , France

## Abstract

Straightforward access to a new cyanide-bridged {Fe_4_Co_4_} “molecular box” containing a potassium ion, namely K⊂{[Fe^II^(Tp)(CN)_3_]_4_[Co^III^(^pz^Tp)]_3_[Co^II^(^pz^Tp)]} (**1**) (with Tp and ^pz^Tp = tris- and tetrakis(pyrazolyl)borate, respectively), is provided, alongside its full characterisation. A detailed analysis of the molecular structure (X-ray diffraction, mass spectrometry, NMR spectroscopy) and electronic properties (EPR spectroscopy, SQUID magnetometry, UV/Vis spectroscopy, cyclic voltammetry) reveals that **1** shows slow magnetic relaxation and a remarkable photomagnetic effect at low temperature which is reminiscent of some FeCo Prussian Blue Analogues (PBAs), and is ascribed to a photo-induced electron transfer. However, in contrast with these inorganic polymers, the overall neutral compound **1** is soluble and remarkably stable in organic solvents such as CH_2_Cl_2_. Moreover, **1** shows interesting redox versatility, with electrochemical experiments revealing the possible access to six stable redox states.

## Abstract 2

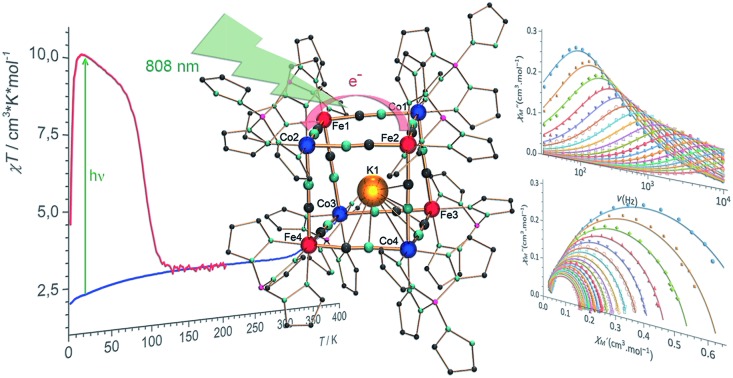
We report a new K⊂[Fe_4_Co_4_] cyanide box: a true soluble model of the photomagnetic FeCo Prussian blue analogues, which also shows photo-switchable SMM properties and remarkable redox versatility.

## Introduction

Switchable molecular systems featuring electronic, magnetic, or optical bistability are attracting strong research interest because of their potential use as molecular memories, switches, actuators, or sensors, and are therefore showing promise in emerging fields such as molecular electronics.^1^–^3^ Cyanide coordination chemistry has proven successful in providing access to a variety of responsive systems, whose optical and magnetic properties can be reversibly switched.^4^,^5^ A representative example is the photomagnetic FeCo Prussian Blue Analogues (PBA) of the composition K_0.2_Co_1.4_[Fe(CN)_6_]·6.9H_2_O, which was first described by O. Sato *et al.*^6^ In this compound, light irradiation can induce an Electron Transfer Coupled to a Spin Transition (ETCST), which thus converts the diamagnetic {FeIILS–CN–CoIIILS} pairs into paramagnetic (FeIIILS–CN–CoIIHS) ones (LS: low spin, HS: high spin), leading to important changes in both optical and magnetic properties. The physical properties of these FeCo PBAs are however highly dependent on their chemical composition. In particular, the amount and the nature of the inserted alkali ions appears to play a crucial role in the occurrence of magnetic and optical bistability.^7^,^8^ In recent years, intense research efforts have been devoted to the synthesis of lower dimensional models of the FeCo PBAs. Polynuclear FeCo complexes or one-dimensional photomagnetic systems have been studied.^9^–^16^ The cyanide-bridged, cubic-shaped {Fe_4_Co_4_} compound reported by Holmes, Clérac, and Mathonière *et al.* in 2008 is a remarkable example of a photomagnetic entity exhibiting a notably long metastable life-time.^9^ Since then, no other FeCo photomagnetic cubes have been reported, but several similar cubic systems showing interesting magnetic properties or redox flexibility have been reported.^17^–^22^ The interest in these systems also originates from their possible use as molecular sensors for the selective binding of alkali ions.^23^,^24^ In this work, we present a novel mixed-valence {Fe_4_Co_4_} molecular box encapsulating a potassium ion. The overall neutral complex shows a photomagnetic effect and single molecule magnet behaviour in the solid state. Besides, it exhibits remarkable redox flexibility in dichloromethane solution with six accessible redox states.

## Results and discussion

### Synthesis and solid state structure of **1**

**Scheme 1:**
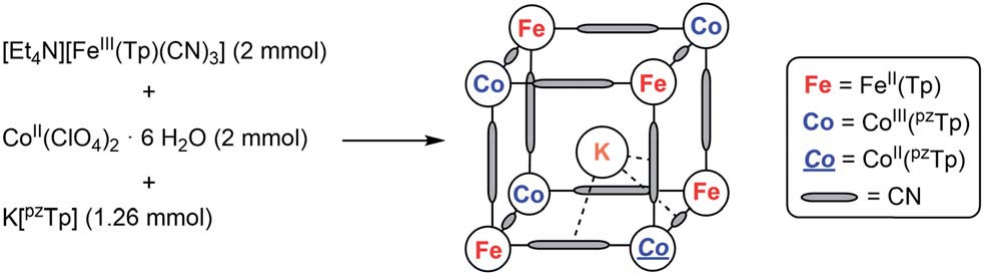
Simplified synthesis of **1** (further details, see ESI‡).

The mixed-valence {Fe_4_Co_4_} heterocubane of the formula K⊂{[Fe^II^(Tp)(CN)_3_]_4_[Co^III^(^pz^Tp)]_3_[Co^II^(^pz^Tp)]} (**1**) (where Tp and ^pz^Tp stand for tris- and tetrakis(pyrazolyl)borate, respectively) is obtained by reacting [Et_4_N][Fe^III^(Tp)(CN)_3_] with Co^II^(ClO_4_)_2_·6H_2_O and K[^pz^Tp] (Scheme 1).

**Fig. 1:**
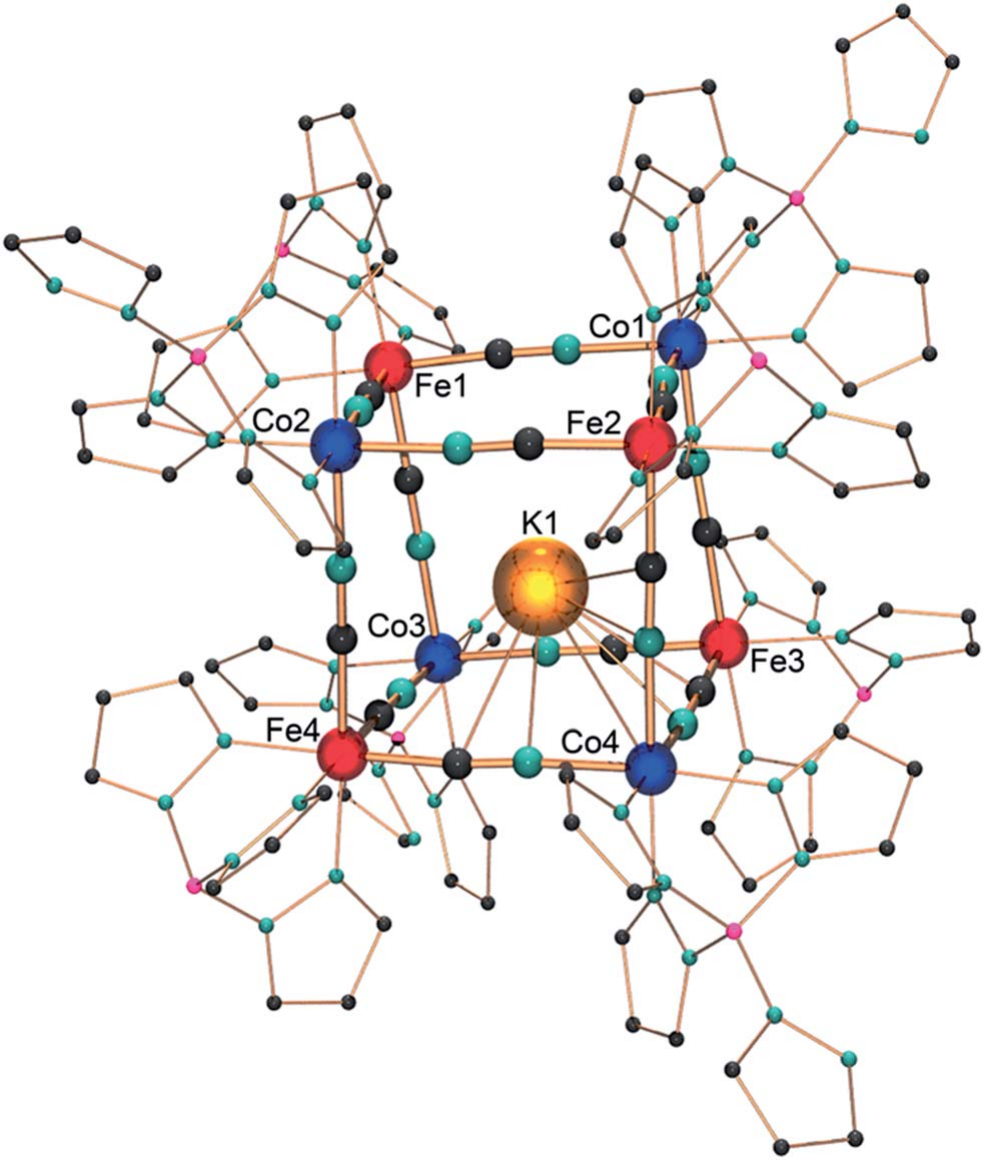
Ball-and-stick representation of the molecular structure of **1** with the highlighted heterocubane core structure K⊂{Fe_4_Co_4_} of **1**. Tp and ^pz^Tp ligands are drawn as spheres with a small radius for clarity; solvent molecules and two of the three partially occupied potassium positions are omitted for clarity (K1: 50% occupancy). Color code: Co: blue; Fe: red; K: orange; C: grey; N: turquoise; B: pink.

Single crystals suitable for X-ray diffraction were obtained by the slow diffusion of pentane in a dichloromethane solution of **1**. Under these conditions, a triclinic crystalline phase is obtained (*P*1[combining macron], *Z* = 2) whose structure is composed of K⊂{Fe_4_Co_4_} cubic motifs (Fig. 1) and dichloromethane lattice molecules (12 per cubic unit). Selected bond lengths are listed in the ESI.‡

Within the {Fe_4_Co_4_} core structure, the iron and cobalt ions occupy alternate corners of the heterocubane, which possesses cyanide ligands in bridging positions along the cube edges. The Fe–CN–Co edge lengths are almost identical, averaging 4.99 Å. The Co–N–C and Fe–C–N angles are only slightly bent (from 171.1(3)° to 178.8(3)°, and from 174.1(3)° to 178.4(3)°, respectively). The four iron ions exhibit very similar octahedral C_3_N_3_ environments, composed of three N donor atoms of the *fac*-coordinated Tp ligands and three cyanide carbon atoms. The Fe–C bond lengths fall in the range of 1.866(4)–1.900(4) Å and are similar to those previously reported for other {Fe^II^(Tp)(CN)_3_} structural motifs.^9^,^14^ The cyanide stretching vibration at 2103 cm^–1^ also supports the occurrence of Fe^II^ ions (Fig. S1‡).^9^,^14^ The four cobalt ions exhibit an octahedral N_6_ environment, surrounded by three N atoms of the *fac*-^pz^Tp ligand and three N atoms of the cyanide ligands. Three cobalt ions (Co1, Co2, Co3, Fig. 1) show shorter Co–N bond lengths, ranging from 1.915(3) to 1.943(4) Å. These values are only slightly longer than those previously observed for low-spin Co^III^ ions in related molecular squares and cubes (*ca.* 1.89–1.91 Å).^9^–^14^ However, they are far shorter than typical Co–N bond lengths, *ca.* 2.1 Å, observed in high-spin cobalt(ii) complexes.^9^,^10^,^12^–^14^ The Co1, Co2, and Co3 coordination spheres also exhibit a moderate octahedral distortion (the sum of the deviation to 90° being *Σ* = 14.7–18.3°), which matches better with low-spin Co^III^ ions. In contrast, the unique cobalt ion Co4 exhibits much longer Co–N distances (average: 2.01 Å), though shorter than typical CoIIHS–N ones, and a significantly more distorted octahedral environment (*Σ* = 28.6°). This is rather indicative of a high-spin Co^II^ ion. Although the Co–N–C angles are slightly bent, they do not show a clear trend or difference between the Co^II^ and Co^III^ ions. Overall, some structural disorder in the position of the CoIIHS and CoIIILS ions likely account for the slight deviations observed in the coordination sphere geometries compared to “usual” CoIIILS and CoIIHS geometries. This seems coherent with the localization of the potassium ion,^25^ which appears to be disordered over different positions inside the cubic cage. As previously observed by Rauchfuss *et al.* in a related K⊂{Rh_4_Mo_4_} cyanide box, the K^+^ Lewis acid is not located in the center of the cube, but establishes interactions with three cyanide π systems, with short K–C and K–N distances of ∼3.2–3.4 Å.^26^ Here, the potassium ion is displaced toward the {Co(NC)_3_} corners but exhibits a marked preference for the Co4 corner (occupancy 50%). It appears reasonable to assume that this preference for the {Co4(NC)_3_} corner correlates with the formal negative local charge of the latter. Despite our efforts to synthesise better-ordered crystal phases, the structural disorder seems to be a marked trend in these cubic systems.

### Magnetic measurements and EPR spectroscopic studies

**Fig. 2:**
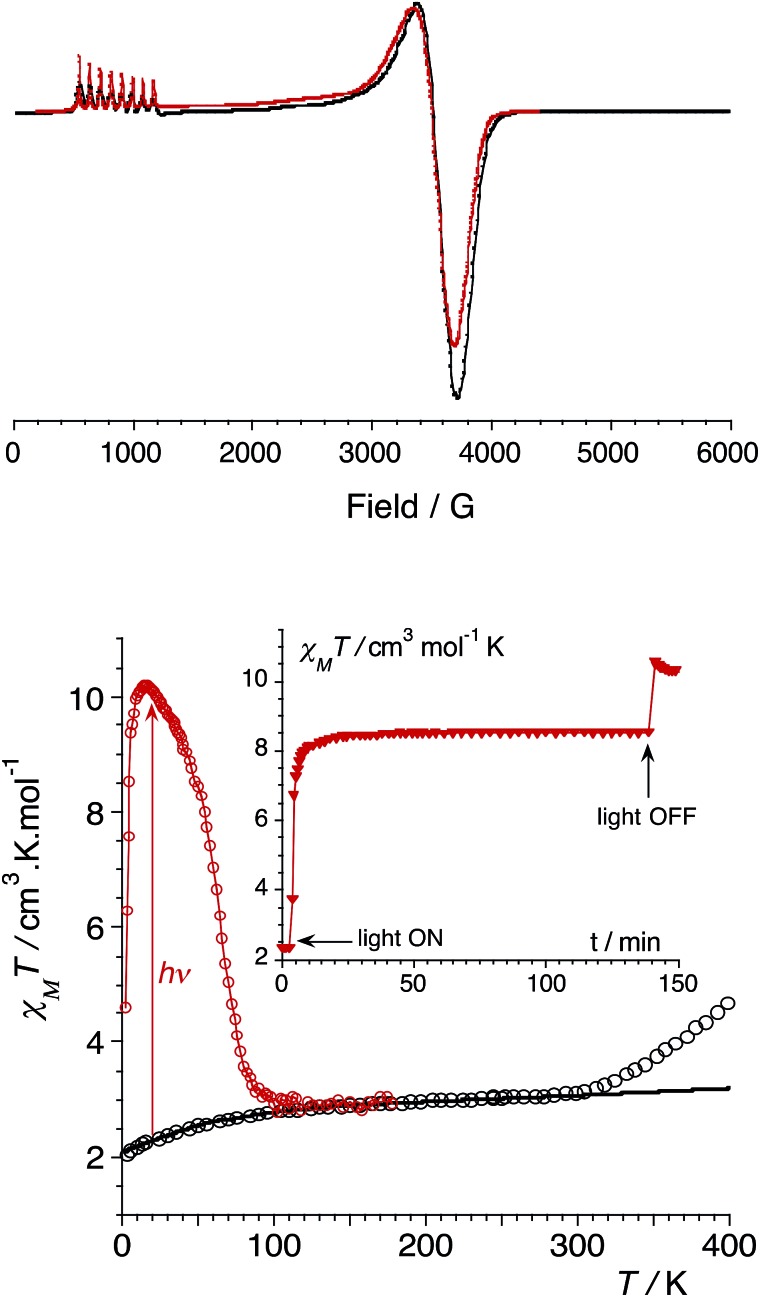
(top) CW X-band EPR powder spectrum of **1** (9.34 GHz) at 4 K (experimental in black and simulated in red). (bottom) Graph of the *χ*_M_*T versus T* plot of **1**: on the bulk upon heating (empty circles, best fit curve in black, see ESI‡); after irradiation at 808 nm (red circles). Inset: time dependence of *χ*_M_*T* at 20 K under irradiation at 808 nm (5 mW cm^–2^).

In order to confirm the electronic states of the metal ions in **1**, we have performed solid state magnetic studies. The continuous-wave X-band EPR spectrum recorded at low temperature (Fig. 2) is typical for octahedral CoIIHS complexes with axial symmetry and a large positive zero-field splitting (*D* [double greater-than, compressed] 9.34 GHz). In such a case, EPR transitions are only observed within the lowest Kramer doublet (with an effective electron spin of 1/2).^27^,^28^ The simulation of the spectrum^29^ leads to the spin-Hamiltonian parameters: *g*_eff⊥_ = 1.85 and *g*_eff‖_ = 7.71, with hyperfine coupling to the CoIIHS ion (*I* = 7/2) of *A*_⊥_ = 80 MHz and *A*_‖_ = 960 MHz. These values are comparable to those observed for octahedral CoIIHS complexes exhibiting, as in the present case, a *C*_3_ axis and containing related tris(pyrazolyl)borate or tris(pyrazolyl)methanide ligands.^30^,^31^

Magnetic susceptibility measurements were performed in the 2–400 K temperature range on freshly filtered crystalline powders (Fig. 2). Up to room temperature, **1** exhibits a *χ*_M_*T vs. T* curve (*χ*_M_ is the molar magnetic susceptibility per cubic unit) which can be analysed considering the presence of one isolated octahedral high-spin Co^II^ ion per cubic unit.^32^ Indeed, the magnetic data between 10 and 300 K can be simulated using the T-P isomorphism approach^33^–^35^ and the following Hamiltonian, which is appropriate to describe isolated Co^II^ ions:

*ℋ*_tot_ = *ℋ*_so_ + *ℋ*_dist_ + *ℋ*_Ze_

with the following contributions:
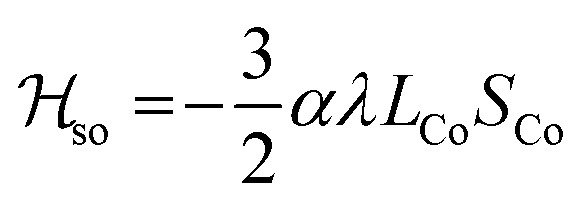
 
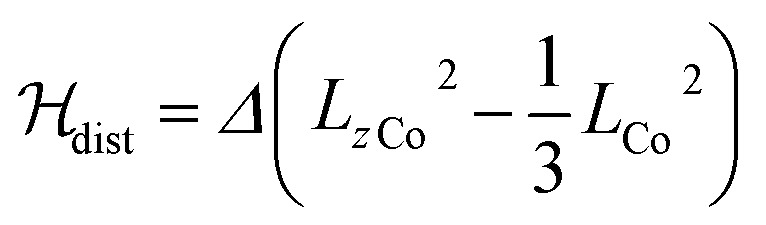
 
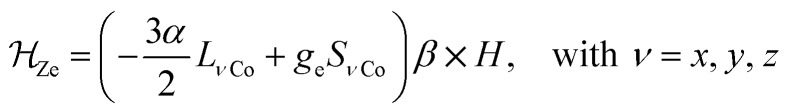
 *λ* is the spin–orbit coupling constant, *α* is the orbital reduction factor and *Δ* is the axial distortion parameter. *L* and *S* are the orbital and spin operators, respectively, with *L* = 1 and *S* = 3/2. The least square fit of the magnetic *χ*_M_*T* data leads to the set of values: *λ* = –147 cm^–1^, *α* = 0.76, and *Δ* = –3638 cm^–1^, with a good agreement factor.^36^

Above approximately 300 K, the *χ*_M_*T vs. T* experimental data deviate from the theoretical curve and show a steady increase that is likely due to a thermally-induced ETCST. In contrast to the previously reported photomagnetic {Fe_4_Co_4_} cube,^9^ which shows a steep thermally-induced transition from the diamagnetic {FeIILSCoIIILS}_4_ state to the paramagnetic {FeIIILSCoIIHS}_4_ state near 250 K, **1** shows a gradual transition. The *χ*_M_*T* values obtained at 400 K and the absence of a plateau indicate a partial ETCST. The measured *χ*_M_*T* value of 4.5 cm^3^ mol^–1^ K at 400 K roughly corresponds to *ca.* 33% of the value expected for the fully paramagnetic state. The shift in the transition temperature is likely associated with the anionic charge and stronger donor character of the ^pz^Tp ligand that stabilises the Co^III^ redox state compared to the related neutral ligand (2,2,2-tris(pyrazolyl)ethanol) used by Holmes *et al.*^9^ It should also be noticed that the thermally-induced ETCST is not reversible after heating the sample up to 400 K (see ESI‡). This is likely associated with the loss of crystallization solvent molecules, as previously observed in other FeCo molecular switches.11*b*

More interestingly, **1** shows significant photomagnetic effects upon irradiation in the visible and near-infrared range. Significant increases in magnetisation are observed upon irradiation with laser light at 405, 532, 635, 808 and 900 nm (Fig. S2, ESI‡). As observed in related {Fe_2_Co_2_} squares,^14^ the 808 nm wavelength is the most efficient, and shows the highest photo-conversion rate. The thermal stability of the photo-induced metastable state was probed by measuring the *χ*_M_*T vs. T* curve after irradiation at 808 nm (empty circles plot in Fig. 2). The metastable state undergoes thermal relaxation at *ca. T*_relax_ = 80 K. This temperature is notably lower than that observed for the only other {Fe_4_Co_4_} photomagnetic cube (*T*_relax_ ≈ 175 K).^9^

**Fig. 3:**
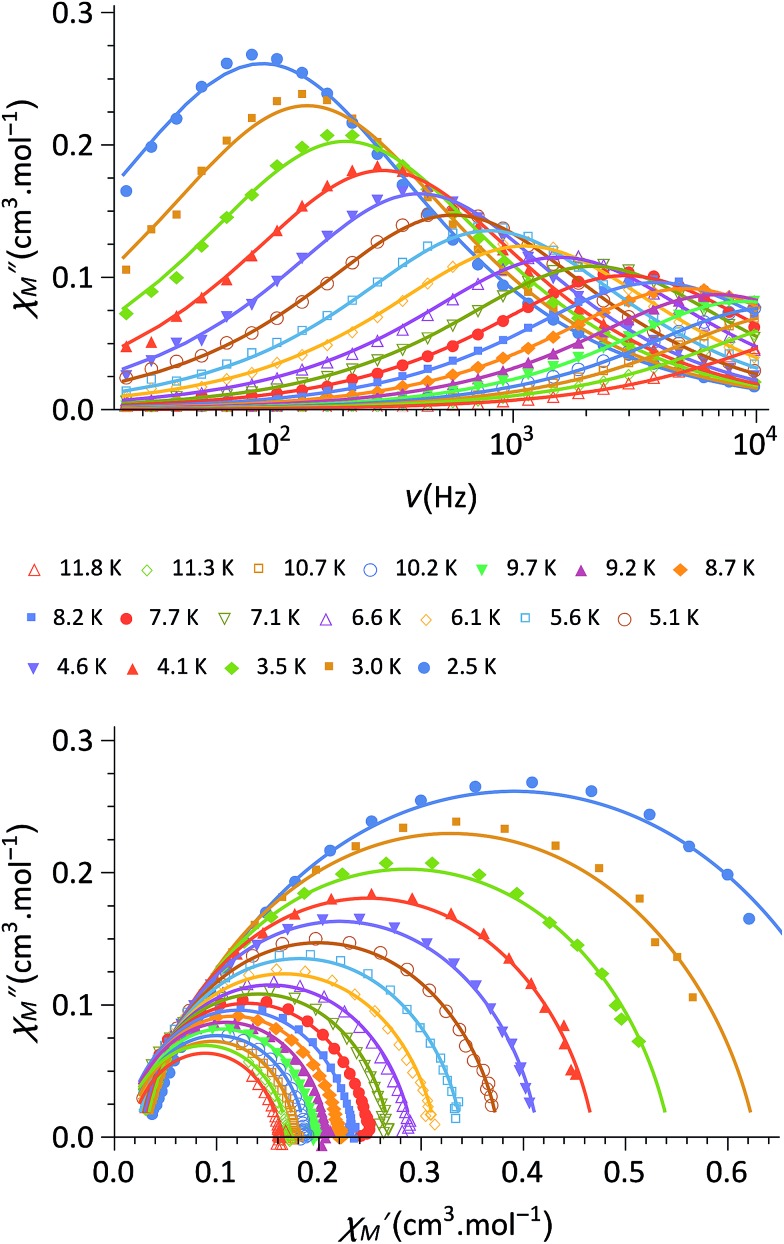
(top) Out-of-phase component, *χ*′′_M_, of the magnetic susceptibility of **1** under a 1.8 kOe magnetic field and for frequencies between 30 Hz and 10 kHz (in phase component in Fig. S4‡); (bottom) Cole–Cole plot of **1** measured from 2.5 to 10 K under 1.8 kOe. The solid lines are the least-squares fitting of the data using a generalized Debye model (see ESI‡).

Finally, the dynamic magnetic properties of **1** were probed by measuring the alternating current magnetic susceptibility as a function of temperature, frequency and dc magnetic field in both the ground state and the photo-induced one (see ESI‡). In the ground-state, out-of-phase susceptibility signals (*χ*′′_M_) were observed under a magnetic field (in the range of 0–3 kOe) with an optimal value of 1.8 kOe (Fig. 3). Only very weak *χ*′′_M_ signals are observed at zero-field (Fig. S4‡), likely because of a fast relaxation through quantum tunnelling of the magnetisation (QTM). The Cole–Cole plots^37^ at 1.8 kOe show a semi-circle shape (Fig. 3) and can be fitted using a generalized Debye model in order to obtain the temperature dependence of the relaxation time *τ*. The linear regime observed in the ln(*τ*) *versus* (1/*T*) curve at high temperature is coherent with an Orbach (thermally-induced) relaxation process (Fig. S5). The fit of the linear region using an Arrhenius law leads to an effective energy barrier for the reversal of the magnetization of 34 cm^–1^ and a relaxation time of *τ*_0_ = 1.1 × 10^–7^ s (see ESI‡). The discrepancies from the linear regime at low temperature are due to other possible relaxation processes such as QTM and Raman relaxation processes. Overall, the complex **1** shows Single-Molecule Magnet (SMM) behaviour, which is reminiscent of that recently observed for other anisotropic Co^II^ complexes.^38^–^40^ It is worth noticing that the slow-relaxation disappears in the photomagnetic state so that **1** could be considered as a light-switchable SMM.

### NMR spectroscopic studies in solution and mass spectrometry

A noticeable feature of **1** is its solubility in organic solvents such as CH_2_Cl_2_. All the solution-based analytical characterisation data point to the remarkable stability of the K⊂{Fe_4_Co_4_} box in CH_2_Cl_2_ solution. For example, the EPR spectrum of **1** in frozen CH_2_Cl_2_ solution is very similar to that obtained for the powder sample, and suggests a similar electronic state (Fig. S6‡).

**Fig. 4:**
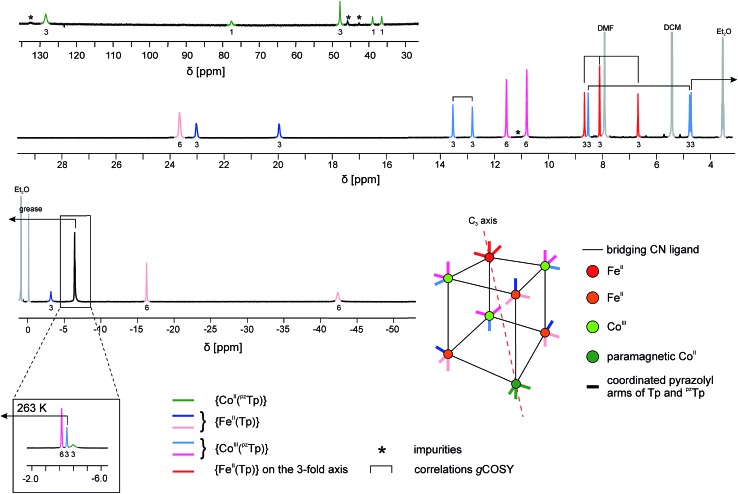
^1^H NMR spectrum of **1** at *T* = 233 K in CD_2_Cl_2_. Since at this temperature three signals overlap at *δ* ∼ –4 ppm, a zoom of the same spectral region at a higher temperature (*T* = 263 K) is depicted in the inset. Partial information about the connectivity within the pyrazolyl rings when precise attribution was not possible (obtained by gCOSY) are depicted as brackets (for more details, see ESI‡).

More convincing structural evidence for the stability of **1** is provided by the NMR study (Fig. 4). The ^1^H NMR spectrum recorded in the 183–293 K temperature range is indeed fully consistent with the ascribed {FeII4CoIII3Co^II^} redox states. The occurrence of one paramagnetic CoIIHS ion in one of the vertices of the cubic unit has several impacts both on the NMR spectrum itself and the interpretation and assignment of the signals. (i) Strongly shifted ^1^H signals are observed in both the positive and negative frequency regions of the spectrum. This effect can help to assign the signals as the chemical shifts are expected to be more important for those protons closely connected to the paramagnetic center.^41^ (ii) Because of the nuclear–electron dipolar interaction, line broadening effects are observed. This interaction depends on the proton–metal distance and also helps to assign the proton signals.^41^ (iii) The chemical shifts show a significant temperature dependence, with an overall increase in the absolute value upon cooling (Fig. S7–S9‡). This behaviour is typical of paramagnetic molecules, as their chemical shifts exhibit a significant contribution from the hyperfine interaction. Here again, the changes in the chemical shifts mainly affect the proton signals located in the vicinity of the paramagnetic ion, bearing a significant amount of spin density.^42^

The occurrence of one non-equivalent Co(ii) ion per cubic unit also induces a lowering of the symmetry from *T*_d_ to *C*_3v_ (assuming that the potassium ion resides on the *C*_3_ axis; see scheme in Fig. 4). Consequently, the pyrazolyl entities of the Tp and ^pz^Tp ligands, whose boron atoms are not located on the *C*_3_ axis, are not equivalent any more. In order to visualise this effect, the non-equivalent pyrazolyl groups are depicted as bars with different colours in the schematic drawing in Fig. 4 (note that the non-coordinated pz groups of the ^pz^Tp ligand are omitted for clarity). Remarkably, all of the 24 expected ^1^H NMR signals of the pyrazolyl entities can be observed in the ^1^H NMR spectrum, provided that the temperature is low enough (in our case, 233 K).^43^ To some extent, the assignment of the pyrazolyl proton signals was either possible due to the above mentioned remarks and using the relative intensity of the signals (see details in the ESI‡), or, for those signals possessing favourable nuclear relaxation, by applying the ^1^H gCOSY method. The ^11^B NMR spectrum recorded at room temperature also supports this structural analysis (Fig. S10‡). Three ^11^B signals are observable, *i.e.* one for the paramagnetic {Co^II^(^pz^Tp)} unit, which strongly shifts with temperature, and two signals at 1.6 ppm and at –13.5 ppm, which can be assigned to the boron atoms of the {Co^III^(^pz^Tp)} units and the {BH} moiety of the {Fe^II^(Tp)} units. Finally, it is worth noticing that the K⊂{Fe_4_Co_4_} box is quite stable in solution over three months, as the NMR spectra recorded during this period only show very small amounts of degradation products (Fig. S11‡).

Diffusion NMR studies were performed on a dichloromethane solution of **1**. In spite of the reduced spin-lattice relaxation times of the proton due to the paramagnetic nature of the complex, the mean diffusion coefficient could be estimated as *D* = 6.88 × 10^–10^ m^2^ s^–1^. This corresponds to a spherical hydrodynamic radius of 7.6 Å, which is in line with the expected value for a fully stable species in solution (see ESI‡).

Mass spectrometric studies provided further proof for the stability of the cubic moieties in CH_2_Cl_2_. In the ESI-MS spectrum (cation mode), a molecular peak corresponding to the mono-oxidized [K⊂{FeII4CoIII4}]^+^ species was observed, with the expected isotopic pattern for an exact molecular mass of *M* = 2779.4 g mol^–1^ (Fig. S13‡). The only other detected peak, at *m*/*z* = 617, corresponds to residual traces of the [Co^III^(^pz^Tp)_2_]^+^ by-product from the last purification step of the synthesis. In the anion mode ESI-MS spectrum, the only detectable signal (*m*/*z* = 2814) corresponds to an adduct between the neutral **1** and a chloride anion from the solvent, furnishing [**1**·Cl]^–^.

### Cyclic voltammetry studies

**Fig. 5:**
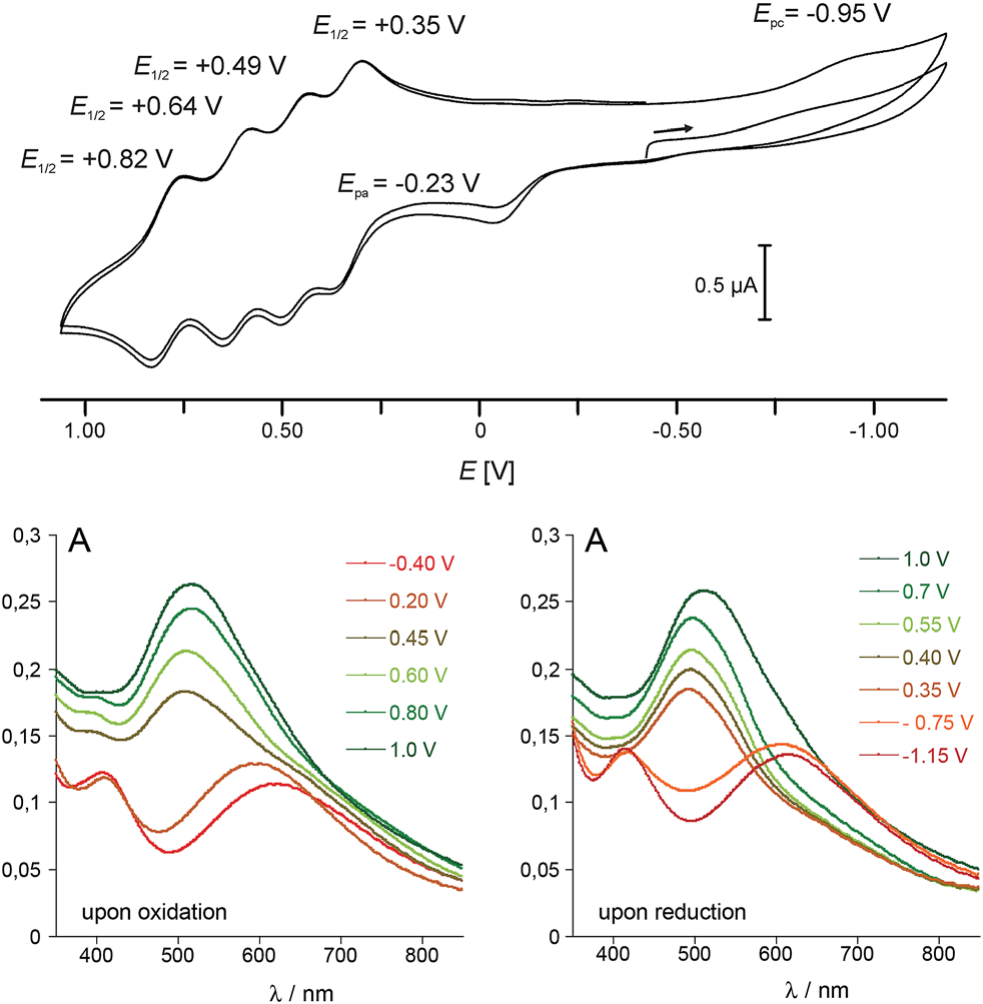
(top) Cyclic voltammogram of **1** in CH_2_Cl_2_ (*vs.* Fc/Fc^+^ (internal standard), [**1**] = 10^–4^ M, 0.05 M [Bu_4_N][PF_6_], scan rate 250 mV s^–1^, Pt/[*n*Bu_4_N][PF_6_]/Ag); (bottom) electronic absorption spectra of **1** obtained at controlled potential ([**1**] = 10^–4^ M, 0.1 M [*n*Bu_4_N][PF_6_] in CH_2_Cl_2_, Pt counter and working electrodes, *vs.* Fc/Fc^+^).

In view of the structural integrity of **1** in organic solvents and in order to probe its redox properties, cyclic voltammetry studies were performed in CH_2_Cl_2_ at room temperature. Interestingly, **1** presents six accessible redox states (Fig. 5). The first redox process at *E*_pa_ = –0.23 V (*vs.* Fc/Fc^+^) is ascribed to the one-electron oxidation of the high-spin Co(ii) centre into a low-spin Co(iii). The corresponding reduction wave is evidenced at *E*_pc_ = –0.95 V (see the ESI‡ for further details). The large difference between the two half waves (*ca*. 0.93 V) is due to the structural reorganisation that accompanies the spin-state change. Such behaviour is typical for a redox process which is coupled to a spin transition and has already been observed for related cobalt complexes.^31^,^44^ At more positive potential values, four well-separated, quasi-reversible redox processes are observable, which are tentatively assigned to the following redox couples:*E*01/2 = +0.35 V: {FeII4CoIII4}/{Fe^III^FeII3CoIII4}^+^*E*01/2 = +0.49 V: {Fe^III^FeII3CoIII4}^+^/{FeIII2FeII2CoIII4}^2+^*E*01/2 = +0.64 V: {FeIII2FeII2CoIII4}^2+^/{FeIII3Fe^II^CoIII4}^3+^*E*01/2 = +0.82 V: {FeIII3Fe^II^CoIII4}^3+^/{FeIII4CoIII4}^4+^

It is worth noting that the coordination of metal ions on the cyanide nitrogen atoms of the [Fe^II/III^(Tp)(CN)_3_]^2–/–^ building block has a strong influence on the Fe^II/III^ oxidation potential. In **1**, this oxidation is shifted by 1.2–1.7 V compared to that of the [Fe^II/III^(Tp)(CN)_3_]^2–/–^ complex (see ESI‡). This behaviour is similar to that observed for some of the previously reported cubic cyanometallate cages (including {Fe_4_Fe_4_}, {Fe_4_Ni_4_} and {Fe_4_Re_4_} cages).^20^–^22^ The redox potential difference between the four Fe^II^ oxidations (Δ*E*) gives access to the respective stability of the mixed-valence states. This can be expressed by the comproportionation constants *K*_c_, with Δ*E* values of 0.14, 0.15 and 0.18 V leading to *K*_c_ values of 2.43 × 10^2^, 3.07 × 10^2^ and 1.03 × 10^3^ for the {Fe^III^FeII3CoIII4}^+^, {FeIII2FeII2CoIII4}^2+^ and {FeIII3Fe^II^CoIII4}^3+^ states, respectively. These values are of the same order of magnitude as those found for the octanuclear, mixed-valence {Fe_4_Fe_4_} complex mentioned above.^20^

Differential absorption spectra of **1** recorded under controlled potential (from –0.40 to 1.00 V and from 1.00 to –1.15 V) were measured to follow the changes in the optical properties which accompany the redox changes (Fig. 5, bottom). Before oxidation, two intense absorption bands are observed in the visible range. The absorption at *λ* = 410 nm (*ε*_410_ = 3585 L mol^–1^ cm^–1^) compares well with that observed at *λ* ≈ 405 nm in the [Fe^II^(L)(CN)_3_]^2–^ complexes (L = Tp and ^pz^Tp), and is ascribed to a metal-to-ligand charge transfer transition (MLCT). The very broad and intense band centred at *λ* = 618 nm (*ε*_618_ = 3500 L mol^–1^ cm^–1^) is reminiscent of that observed around 550 nm in K_*x*_[Co_*y*_[Fe(CN)_6_]_*z*_ PBAs,^6^,^45^ and is assigned to a Fe^II^–Co^III^ charge transfer transition (MMCT). Upon oxidation of the Co^II^ ion, from –0.4 to +0.2 V, a blue shift of the band ascribed to the Fe^II^–Co^III^ CT transition is observed, together with an increase in its intensity (the absorption shifts from 620 nm to *ca.* 590 nm). As the potential is further increased to oxidize the four Fe^II^ ions, a significant increase in the intensity of the Fe-centred MLCT band (near *ca.* 500 nm) is observed, as previously reported for the related {Fe_4_Fe_4_} complex.^20^ The phenomenon appears to be fully reversible as the successive reduction of the four Fe(iii) ions induces a decrease of this band. The initial state is then recovered near *ca.* –1.1 V in the present experimental conditions as the low-spin Co^III^ ion requires a much lower potential to be reduced (see cyclic voltammetry studies above). However, if the strongly reductive potential is maintained for a few minutes at this stage, slow decomposition of the cube can be observed.

## Conclusions

The first cyanide-bridged K⊂{Fe_4_Co_4_} “molecular box” (**1**) containing an inserted potassium ion is reported. As with the original K_0.2_Co_1.4_[Fe(CN)_6_]·6.9H_2_O inorganic polymer, **1** exhibits a remarkable photomagnetic effect at low temperature, which is ascribed to a photo-induced electron transfer. Remarkably, the complex also behaves as a photo-switchable single molecule magnet. As evidenced by various methods such as NMR and EPR spectroscopy, ESI-MS spectrometry, and electrochemistry, compound **1** is soluble and stable in CH_2_Cl_2_ solution. Indeed, the overall neutral complex remains fully undissociated in solution, still possessing the potassium-filled heterocubane-type structure. Moreover, cyclic voltammetry studies reveal that **1** exhibits remarkable redox flexibility with six accessible redox states. The stability and the electronic flexibility of **1** are appealing features, which open up perspectives for the insertion of this switchable molecule into hybrid materials. Our current efforts are devoted to more fundamental aspects, as we are currently exploring the role of the nature of the inserted alkali ions on the electronic and magnetic properties of similar “molecular boxes”.

## Supplementary Material

Supplementary information

Crystal structure data
