# Supplementary material for: K⊂{[FeII(Tp)(CN)3]4[CoIII(pzTp)]3[CoII(pzTp)]}: a neutral soluble model complex of photomagnetic Prussian blue analogues
Source: Chem Sci. 2016 May 13;7(8):4825–31. doi: 10.1039/c6sc01435f (PMC6014070; doi:10.1039/c6sc01435f)
Supplement: Supplementary file 1 [file SC-007-C6SC01435F-s001.pdf]

Electronic Supplementary Material (ESI) for Chemical Science.
This journal is © The Royal Society of Chemistry 2016

Electronic Supplementary information (ESI)

The neutral complex K⊂{[Fe^{II}(Tp)$(CN)_3$]$_4$[Co^{III}(^{pz}Tp)]$_3$[Co^{II}(^{pz}Tp)]}: a soluble model of photomagnetic Prussian blue analogues

D. Garnier,[a,b] J. R. Jiménez,[b] Y. Li,[b] J. von Bardeleben,[c] Y. Journaux,[b] T. Augenstein,[a] E. Moos,[a] M. T. Gamer,[a] F. Breher*,[a] and R. Lescouëzec*,[b]

[a] Institute of Inorganic Chemistry, Karlsruhe Institute of Technology (KIT), Engesserstr. 15, Geb. 30.45, D-76131 Karlsruhe, Germany, E-mail: breher@kit.edu

[b] Institut Parisien de Chimie Moléculaire - CNRS UMR 8232, UPMC - Paris 6, Sorbonne Universités, 4 place Jussieu, F-75252 Paris cedex 05 France, E-mail: rodrigue.lescouezec@upmc.fr

[c] Institut des Nanosciences de Paris - CNRS UMR 7588, UPMC – Paris 6, Sorbonne Universités, 4 place Jussieu, F-75252 Paris cedex 05 France.

TOC of the Supporting Information:

Section S1 – Experimental and technical details S3
S1.1 – Synthetic procedure S3
S1.2 – Technical details S3
Section S2 – Crystallographic details and bond lengths and angles S7
Section S3 – FT-IR spectrocopy S10
Section S4 – Magnetic measurements and EPR spectroscopic studies S11
Section S5 – NMR spectroscopy S14
Section S6 – Mass spectrometry S23
Section S7 – Cyclic voltammetry S27
Section S8 – UV-visible absorption spectra S30

Section S1 – Experimental and technical details

S1.1 – Synthetic procedure

Reagents were purchased from Aldrich or Acros and used without further purification. $[Et_4N][Fe^{III}(Tp)(CN)_3]$ was synthesised according to the procedure reported by Lescouëzec *et al.*,[S1] changing the $[PPh_4]Cl$ salt by $[NEt_4]Cl$.

To a stirred yellow solution of $[Et_4N][Fe^{III}(Tp)(CN)_3]$ (0.954 mg, 2.0 mmol) in 10 mL DMF was added solid $Co^{II}(ClO_4)_2 \cdot 6\ H_2O$ (732 mg, 2.0 mmol). The red resulting solution was stirred for 20 minutes. It was precipitated with 100 mL Et_2O and the supernate was removed. 580 mg of the red powder were dissolved in 14 mL of DMF and *ca.* 6.3 equivalents of solid $K^{pz}Tp$ (400 mg, 1.26 mmol) were added. The stirred red solution immediately turned deep green and was further stirred overnight. The resulting suspension was centrifugated and the yellow supernate was removed. The obtained blue solid was washed several times with an Et_2O/DMF mixture (v/v 8:1) until the supernate was colourless. Subsequently, the solid was dissolved in diethyl ether and filtrated to remove an off-white solid. Ether was evaporated and **1** was recrystallised either by layering a CH_2Cl_2 solution of **1** with pentane. Yield (as powder): 342 mg (58 %).

Elemental analysis (%): calculated for $C_{96}H_{88}N_{68}B_8Co_4Fe_4K \cdot 4.5\ H_2O \cdot 1\ C_3H_7NO$ (dried in air): C 40.66, H 3.55, N 33.05; found: C 40.54, H 3.42, N 32.94.

[S1] R. Lescouëzec, J. Vaissermann, F. Lloret, M. Julve, M. Verdaguer, *Inorg. Chem*. **2002**, *41*, 5943-5945.

S1.2 – Technical details

FT-IR spectra were collected in the 200-4000 cm^{-1} range. Measurements were carried out on a Vertex 70 Bruker instrument using the attenuated total reflection (ATR) technique on solid samples (with a 4 cm^{-1} resolution). The intensity of the absorption band is indicated as vw (very weak), w (weak), m (medium), s (strong), vs (very strong), and br (broad).

NMR spectra were recorded on Bruker Avance 300 and 400 spectrometers; chemical shifts, δ, are expressed in parts per millions (ppm) and are referenced using IUPAC recommendations. For broad ^1H NMR signals, the full-width-at-half-maximum values ($\omega_{1/2}$) are given in Hz. Low temperature experiments were performed on calibrated spectrometer with a 4% methanol in MeOD-d_4 sample for temperature corrections. The measurements of the paramagnetic samples were carried out after thermal stabilization. All diffusion processing and molecular size estimation were performed using the DiffAtOnce software package available at www.diffatonce.com. Gradients of the SMSQ10.100 form were used and were calibrated using HDO in D_2O ($D = 1.902\cdot10^{-9}\ m^2\cdot s^{-1}$) prior to measurement. Absence of convection was checked using different measurements with different diffusion times Δ (ms).

Mass spectrometric analyses (ESI mode) were performed on a quadrupole-Orbitrap Q Exactive mass spectrometer from Thermo Scientific.

Elemental analyses (C, H, N, S) were carried out by combustion analysis using a vario MICRO cube apparatus from Elementar.

X-ray crystallography: Crystal data and details concerning data collection and refinement are given below. Data collection was performed on a STOE STADIVARI diffractometer using graphite-monochromated Mo K_{α} radiation with λ = 0.71073 Å. Using Olex2,[S2] the structure was solved with the ShelXT [2] structure solution program using Direct Methods and refined with the ShelXL [3] refinement package using Least Squares minimisation. Structure solution for **1** was by dual-space direct methods with SHELXT[S3] with full-matrix least-squares refinement using SHELXL-2014/7.[S4] All non-hydrogen atoms were refined anisotropically. H atoms were placed in calculated positions using a riding model. All 12 CH_2Cl_2 crystal lattice solvent molecules in the asymmetric unit of **1** could be refined, but showed severe disorder even after modelling of multiple splitted molecules. So the electron density found in the solvent-accessible regions was removed via the PLATON SQUEEZE tool,[S5] which calculates the solvent contribution to the structure factors by back-Fourier transformation. The potassium position was found to be disordered over at least 4 positions with finally refined occupations of 50, 20 (2x) and 10%. Upon convergence, the final Fourier difference map of the X-ray structures showed no significant peaks. Crystallographic data (excluding structure factors) for the structure in this paper has been deposited with the Cambridge Crystallographic Data Centre as supplementary publication no. CCDC 1469358. Copies of the data can be obtained, free of charge, on application to CCDC, 12 Union Road, Cambridge CB2 1EZ, UK: https://summary.ccdc. cam.ac.uk/structure-summary-form.

1 · 12 CH_2Cl_2 (CCDC 1469358): $C_{108}H_{112}B_8N_{68}OCl_{24}Co_4Fe_4K$ (M =3798.16 g/mol): triclinic, space group P-1 (no. 2), a = 1640.51(5) pm, b = 1745.87(5) pm, c = 2983.13(8) pm, α = 84.336(2)°, β = 81.254(2)°, γ = 71.749(2)°, V = 8008.0(4) 10^6 pm^3, Z = 2, T = 100 K, $\mu(MoK_{\alpha})$ = 0.839 mm^{-1}, D_{calcd} = 1.153 g/cm^3, crystal dimensions (*ca.*): 0.433 × 0.351 × 0.142 mm^3, 67140 reflections measured (2.636° ≤ 2Θ ≤ 52.37°), 31472 unique (R_{int} = 0.0658, R_{sigma} = 0.0912) which were used in all calculations. 1873 parameters, 279 restraints. The final R_1 was 0.0527 (I > 2σ(I)) and wR_2 was 0.1243 (all data), max/min residual electron density: 0.943/-0.571 e 10^{-6} pm^{-3}.

[S2] O.V. Dolomanov, L.J. Bourhis, R.J. Gildea, J.A.K. Howard, H. Puschmann, *J. Appl. Cryst.*, **2009**, 42, 339-341.

[S3] G. M. Sheldrick, *Acta Cryst.*, 2015, **A71**, 3-8.

[S4] G. M. Sheldrick, *Acta Cryst.*, 2015, **C71**, 3-8.

[S5] A.L. Spek, *Acta Cryst*. **2009**, D65, 148-155; A.L. Spek, *Acta Cryst*., **2015**, C71, 9-18.

DC and AC Magnetic susceptibility measurements were carried out using respectively a Quantum Design SQUID magnetometer and a PPMS-9 susceptometer on a polycrystalline powder that was filtered from the mother solution just before the SQUID magnetometry experiment. The DC measurements were performed in the temperature range 2 - 400 K under a magnetic field of 0.1 T (20 - 400K) and 0.05 T (40 - 2K), the AC measurements were carried out in the temperature range 2.5-15 K under an oscillating field of 10 Oe within the frequency range 10 - 9900 Hz, under static fields of 0 and 1800 Oe. The magnetic susceptibility values were corrected from the diamagnetism of the molecular constituents and of the sample holder. Magnetic fields of 0.1 T (20-400 K) and 0.05 T (40-2 K) were used for measuring the $\chi_M T$ *vs* *T* curves. The sample (packed in a polyethylene bag) was introduced in the SQUID at 200 K under helium flow and frozen before purging under vacuum. The susceptibility measurements were carried out from 200 to 2 K, then from 2 K to 400 K, and finally from 400 to 2 K.

The Cole-Cole plots have been modelled using generalized Debye model,[S6] which considers a distribution of relaxation time. As suggested by Dekker *et al.,*[S7] two steps procedures were used to extract the relaxation time τ at each temperature. First a fit to the locus of χ of the Cole=Cole Plot was performed to extract the values of χ_{adia}, χ_{iso} and α using the following equation independant of τ and of the frequency.

$$\chi'' = \frac{1}{4\cos^2\left(\frac{\pi\alpha}{2}\right)}\left(\sqrt{2}\sqrt{\cos^2\left(\frac{\pi\alpha}{2}\right)\left(-\cos(\pi\alpha)(\chi_{adia}+\chi_{isoT}-2\chi')^2+4\chi'(\chi_{adia}+\chi_{isoT})-6\chi_{adia}\chi_{isoT}+\chi_{adia}^2+\chi_{isoT}^2-4(\chi')^2\right)}+\sin(\pi\alpha)(\chi_{adia}-\chi_{isoT})\right)$$

where χ_{adia} and χ_{iso} stand for adiabatic and isothermal susceptibilties respectively and α characterizes the spreading of the relaxation time. In a second step τ is obtained by the fit of χ' and χ'' using the values of χ_{adia}, χ_{iso} and α determined in the first step:

$$\chi' = \frac{(\chi_{isoT}-\chi_{adia})\left((2\pi)^{1-\alpha}\sin\left(\frac{\pi\alpha}{2}\right)(\nu\tau)^{1-\alpha}+1\right)}{(2\pi)^{2-2\alpha}(\nu\tau)^{2-2\alpha}+2^{2-\alpha}\pi^{1-\alpha}\sin\left(\frac{\pi\alpha}{2}\right)(\nu\tau)^{1-\alpha}+1}+\chi_{adia}$$

$$\chi'' = \frac{(2\pi)^{1-\alpha}\cos\left(\frac{\pi\alpha}{2}\right)(\nu\tau)^{1-\alpha}(\chi_{isoT}-\chi_{adia})}{(2\pi)^{2-2\alpha}(\nu\tau)^{2-2\alpha}+2^{2-\alpha}\pi^{1-\alpha}\sin\left(\frac{\pi\alpha}{2}\right)(\nu\tau)^{1-\alpha}+1}$$

[S6] K. S. Cole, R. H. Cole, *J. Chem. Phys.,* **1941**, *9*, 341-351.

[S7] C. Dekker, A. F. M. Arts, H. W. de Wijn, A. J. van Duyneveldt, J. A. Mydosh, *Phys. Rev. B* **1989**, *40*, 11243-11251.

Photomagnetic measurements were performed on *ca*. 0.4 mg of sample with an homemade sample holder (an optical fiber was inserted into a commercial rod from Quantum Design) and using standard laser diode from LOT-oriel. The fresh sample was introduced in the SQUID at 200 K under a helium flow and frozen before purging in order to avoid solvent loss. The irradiation was performed in the SQUID at 20 K in order to limit the effect of the heating due to the sample irradiation (H = 1 T). The relaxation of the photo-induced metastable state ($\chi_M T$ *vs T* curve) was measured at 0.4 K min^{-1}.

The **EPR spectrum** of a polycrystalline powder sample of **1** was measured at X-band (9.34 GHz) with a Bruker ER200 instrument equipped with a liquid helium cryostat from Oxford, Inc. The temperature was varied between 4 K and 80 K with various microwave powers (0.02 mW- 2mW), and with a modulation amplitude of 10 Gauss and a modulation frequency of 100 kHz.

UV-vis-NIR absorption spectra were recorded on a JASCO V670 spectrometer at room temperature on CH_2Cl_2 solutions of **1** (14.07 μM and 0.1 mM). Controlled potential absorption spectra were done in a 1 mm path length quartz cell at room temperature. An autolab PGSTAT 100 electrochemical analyser was used as a potentiostat. Electrochemical experiments were performed in dichloromethane with 0.1 M $[Bu_4N][PF_6]$ as the supporting electrolyte and by using a three-electrode cell containing: a platinum-mesh working electrode, a platinum wire counter electrode and an $Ag/AgNO_3$ reference electrode. A cyclic voltammogram was first recorded in a standard electrochemical cell and by using a Pt wire as working electrode (to obtain enough resolution) in order to check the redox potentials in these experimental conditions. The measurements UV-vis spectra at controlled potential were then performed with a JASCO V670 spectrometer.

Cyclic voltammetry measurements were carried out at room temperature in a standard cell equipped with a platinum wire counter electrode, Ag/Ag^+ reference electrode and a platinum wire working electrode using Metrohm PGSTAT 101 electrochemical analyzer. The measurements were performed in dichloromethane with 0.05 M $[Bu_4N][PF_6]$ as the supporting electrolyte at scan rates between 50-500 mV s^{-1}. The redox potentials were determined by using ferrocene/ferrocenium couple (Fc/Fc^+) as an internal reference.

Section S2 – Crystallographic details and bond lengths and angles

Table S1. Crystallographic data for **1**.

Compound	**1**·12 CH_2Cl_2
Molecular formula	$C_{108}H_{112}B_8Cl_{24}Co_4Fe_4KN_{68}O$
M [g·mol^{-1}]	3798.16
Crystal system	triclinic
Space group	$P\bar{1}$
a [Å]	16.4051(5)
b [Å]	17.4587(5)
c [Å]	29.8313(8)
α [°]	84.336(2)
β [°]	81.254(2)
γ [°]	71.749(2)
V [Å^3]	8008.0(4)
Crystal size	0.433×0.351×0.142
μ [mm^{-1}]	1.250
$\rho_{calculated}$ [g·cm^3]	1.575
Z	2
T [K]	100
$2\theta_{max}$ [°]	52.37
Collected reflexions	67957
Unique reflexions	31384
Number of parameters/restraints	1873/279
R1 [I ≥ 2σ(I)]	0.0527
wR2 (all data)	0.1243
Max/min residual electron density [e×Å^{-3}]	0.943/-0.571
Radiation	Mo Kα (λ = 0.71073)
Diffractometer	STOE STADIVARI

Table S2. Selected bond lengths for **1**.

Fe1		Fe2		Fe3		Fe4	
Fe1–C1	1.895(4)	Fe2–C4	1.888(4)	Fe3–C7	1.886(4)	Fe4–C10	1.893(4)
Fe1–C2	1.894(4)	Fe2–C5	1.866(4)	Fe3–C8	1.888(4)	Fe4–C11	1.900(4)
Fe1–C3	1.896(4)	Fe2–C6	1.891(4)	Fe3–C9	1.882(4)	Fe4–C12	1.874(4)
Fe1–N101	2.003(3)	Fe2–N201	2.018(4)	Fe3–N301	2.010(3)	Fe4–N401	1.997(3)
Fe1–N111	2.007(3)	Fe2–N211	2.011(4)	Fe3–N311	2.009(4)	Fe4–N411	2.010(3)
Fe1–N121	2.017(3)	Fe2–N221	2.010(8)	Fe3–N321	2.009(3)	Fe4–N421	2.016(4)

Co1		Co2		Co3		Co4	
Co1–N1	1.941(3)	Co2–N2	1.940(4)	Co3–N3	1.916(3)	Co4–N6	1.998(3)
Co1–N5	1.919(4)	Co2–N4	1.943(4)	Co3–N9	1.915(3)	Co4–N7	1.987(4)
Co1–N8	1.929(3)	Co2–N10	1.940(3)	Co3–N12	1.908(4)	Co4–N11	2.003(4)
Co1–N501	1.952(3)	Co2–N601	1.866(7)	Co3–N701	1.912(4)	Co4–N801	2.026(4)
Co1–N511	1.940(3)	Co2–N611	1.943(4)	Co3–N711	1.936(3)	Co4–N811	2.003(4)
Co1–N521	1.958(3)	Co2–N621	1.946(4)	Co3–N721	1.939(3)	Co4–N821	2.020(4)

Table S3. Selected angles around the metal ions of **1**.

Co1		Fe1		Co2	
N5-Co1-N8	87.99(13)	C1-Fe1-C2	88.31(15)	N601-Co2-N4	86.6(3)
N5-Co1-N1	91.62(13)	C1-Fe1-C3	89.49(14)	N4-Co2-N10	90.32(13)
N8-Co1-N1	90.90(12)	C2-Fe1-C3	92.2(2)	N601-Co2-N2	90.2(3)
N5-Co1-N501	89.73(14)	C1-Fe1-N101	92.92(14)	N4-Co2-N2	91.52(14)
N8-Co1-N501	89.73(14)	C3-Fe1-N101	89.98(15)	N10-Co2-N2	88.93(13)
N8-Co1-N511	93.78(14)	C2-Fe1-N111	91.53(15)	N601-Co2-N621	89.9(3)
N1-Co1-N511	90.65(12)	C3-Fe1-N111	90.83(14)	N4-Co2-N621	89.6(2)
N501-Co1-N521	88.30(13)	N101-Fe1-N111	87.23(14)	N10-Co2-N621	90.98(14)
N5-Co1-N521	91.61(13)	C1-Fe1-N121	92.46(13)	N601-Co2-N611	90.9(3)
N1-Co1-N521	90.65(12)	C2-Fe1-N121	90.61(14)	N10-Co2-N611	92.14(13)
N501-Co1-N511	88.14(14)	N101-Fe1-N121	87.18(13)	N2-Co2-N611	91.32(15)
N511-Co1-N521	86.57(14)	N111-Fe1-N121	87.23(13)	N621-Co2-N611	87.6(2)
Fe2		Co3		Fe3	
C5-Fe2-C6	92.74(15)	N9-Co3-N701	88.93(14)	C7-Fe3-C9	87.6(2)
C5-Fe2-C4	87.4(2)	N9-Co3-N3	90.54(13)	C7-Fe3-C8	94.44(15)
C6-Fe2-C4	88.07(15)	N701-Co3-N3	91.91(14)	C9-Fe3-C8	88.06(15)
C6-Fe2-N221	90.5(4)	N9-Co3-N12	91.28(13)	C7-Fe3-N301	91.62(15)
C4-Fe2-N221	95.1(3)	N3-Co3-N12	89.15(13)	C8-Fe3-N301	91.79(14)
C5-Fe2-N201	89.08(15)	N701-Co3-N711	88.0(2)	C7-Fe3-N321	90.30(15)
C4-Fe2-N201	93.86(15)	N3-Co3-N711	91.67(14)	C9-Fe3-N321	93.02(14)
N221-Fe2-N201	87.6(4)	N12-Co3-N711	91.76(14)	N301-Fe3-N321	87.19(13)
C5-Fe2-N211	93.0(2)	N9-Co3-N721	89.44(14)	C9-Fe3-N311	93.33(15)
C6-Fe2-N211	89.94(15)	N701-Co3-N721	88.58(15)	C8-Fe3-N311	87.76(15)
N221-Fe2-N211	84.6(3)	N12-Co3-N721	90.36(14)	N301-Fe3-N311	87.46(14)
N201-Fe2-N211	88.13(15)	N711-Co3-N721	88.38(15)	N321-Fe3-N311	87.48(14)
Co4		Fe4			
N11-Co4-N6	90.81(13)	C12-Fe4-C10	91.83(15)		
N11-Co4-N7	91.18(13)	C12-Fe4-C11	88.33(15)		
N6-Co4-N7	87.67(13)	C10-Fe4-C11	88.39(15)		
N11-Co4-N811	90.41(13)	C12-Fe4-N401	90.62(15)		
N7-Co4-N811	94.51(13)	C10-Fe4-N401	89.95(14)		
N11-Co4-N821	90.73(13)	C12-Fe4-N411	91.31(15)		
N6-Co4-N821	91.06(13)	C11-Fe4-N411	93.94(14)		
N811-Co4-N821	86.71(14)	N401-Fe4-N411	87.77(14)		
N6-Co4-N801	93.18(13)	C10-Fe4-N421	90.58(15)		
N7-Co4-N801	91.91(13)	C11-Fe4-N421	92.61(15)		
N811-Co4-N801	85.49(14)	N401-Fe4-N421	88.51(14)		
N821-Co4-N801	86.26(13)	N411-Fe4-N421	86.24(15)		

Table S4. Selected cyanide bent angles for **1**.

N1-C1-Fe1	176.1(3)	N2-C2-Fe1	177.1(3)	N3-C3-Fe1	177.7(3)
N4-C4-Fe2	174.1(3)	N5-C5-Fe2	175.7(3)	N6-C6-Fe2	178.4(3)
N7-C7-Fe3	177.4(3)	N8-C8-Fe3	174.7(3)	N9-C9-Fe3	175.2(3)
N10-C10-Fe4	178.0(3)	N11-C11-Fe4	174.3(3)	N12-C12-Fe4	178.7(3)
C1-N1-Co1	178.7(3)	C5-N5-Co1	172.7(3)	C8-N8-Co1	173.3(3)
C2-N2-Co2	176.2(3)	C4-N4-Co2	173.4(3)	C10-N10-Co2	176.5(3)
C3-N3-Co3	175.5(3)	C9-N9-Co3	171.1(3)	C12-N12-Co3	178.8(3)
C6-N6-Co4	177.8(3)	C7-N7-Co4	173.4(3)	C11-N11-Co4	175.1(3)

Section S3 – FT-IR spectrocopy

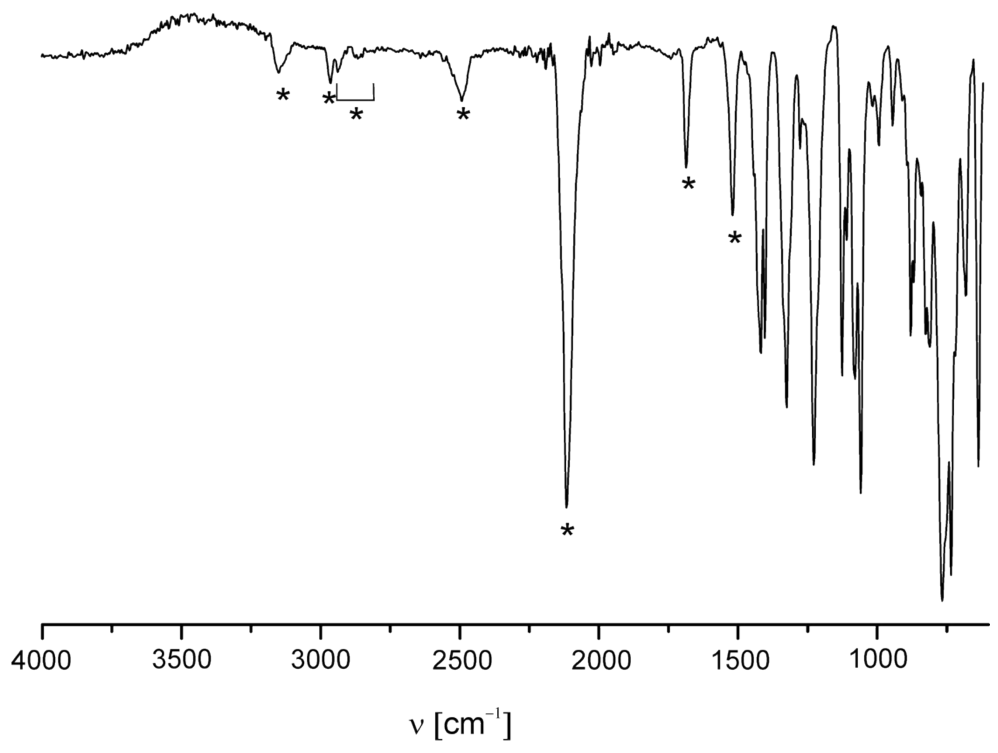

Figure S1. FT-IR (ATR) transmission spectra of fresh crystals of **1** (4 cm^{-1} resolution).

The following selected IR vibration bands in cm^{-1} and their intensities are marked with an asterisk:

pyrazolyl ring breathing (Tp and ^{pz}Tp): 1503 (w),

C-H (pyrazolyl groups of Tp and ^{pz}Tp) 3108 (vw), 3131 (vw), 3146 (vw)

Cyanide stretching#: 2103 (br, m).

B-H stretching (Tp): 2480 (vw),

DMF: 1669 (br, s), 2845 (vw), 2932 (vw),

CH_2Cl_2 : 2960 (vw)

#The cyanide stretching vibration is centred at 2103 but it is remarkably broad and likely results from the superimposition of different contributions.

IR (ATR, ν, cm^{-1}): 401 (w), 431 (w), 446 (w), 484 (m), 514 (s), 548 (w), 618 (vs), 658 (s), 715 (vs), 756 (vs), 801 (s), 825 (w), 850 (s), 860 (m), 926 (w), 976 (w), 1012 (w), 1041 (s), 1059 (s), 1094 (s), 1107 (m), 1152 (vw), 1178 (m), 1207 (s), 1252 (w), 1296 (m), 1307 (s), 1386 (s), 1405 (m), 1430 (w), 1448 (vw), 1503 (w), 1669 (br, s), 2103 (br, m), 2480 (vw), 2845 (vw), 2932 (vw), 2960 (vw), 3108 (vw), 3131 (vw), 3146 (vw).

Section S4 – Magnetic measurements and EPR spectroscopic studies

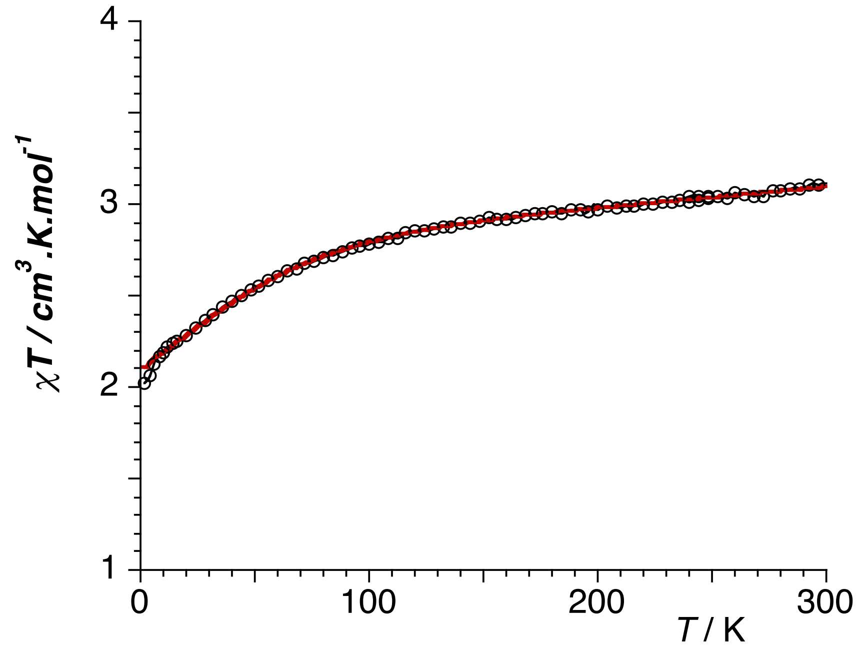

Figure S2a. Zoom of the $\chi_M T$ *vs* temperature curve of **1** in the 0-300 K temperature range (black circles: experimental points; red line: theoretical model).

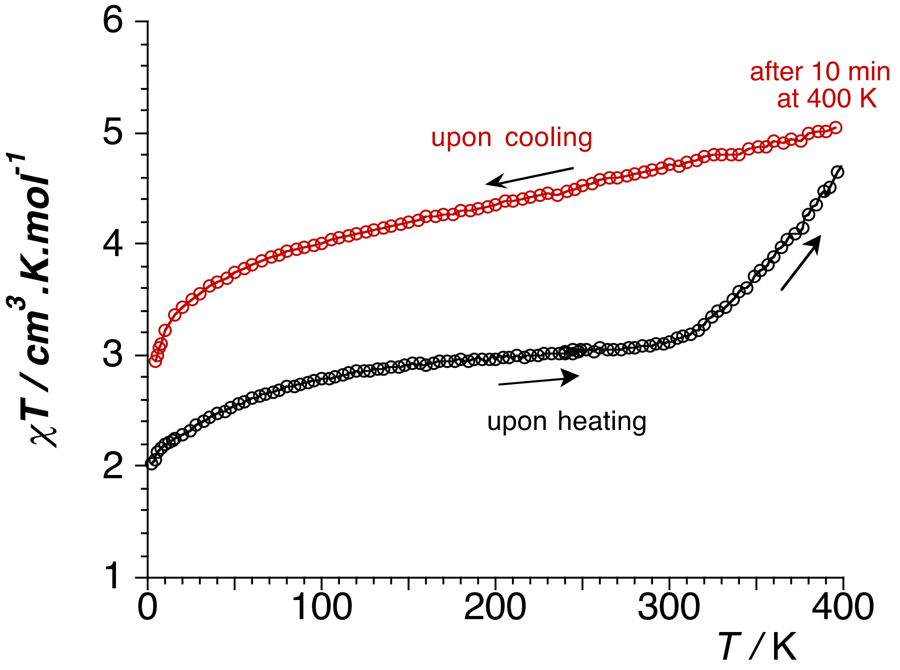

Figure S2b. $\chi_M T$ *vs* temperature curve of **1** measured in the 2 - 400 K temperature range, upon heating (black circles) and upon cooling (red circles) after leaving the sample 10 minutes at 400 K in the SQUID magnetometer.

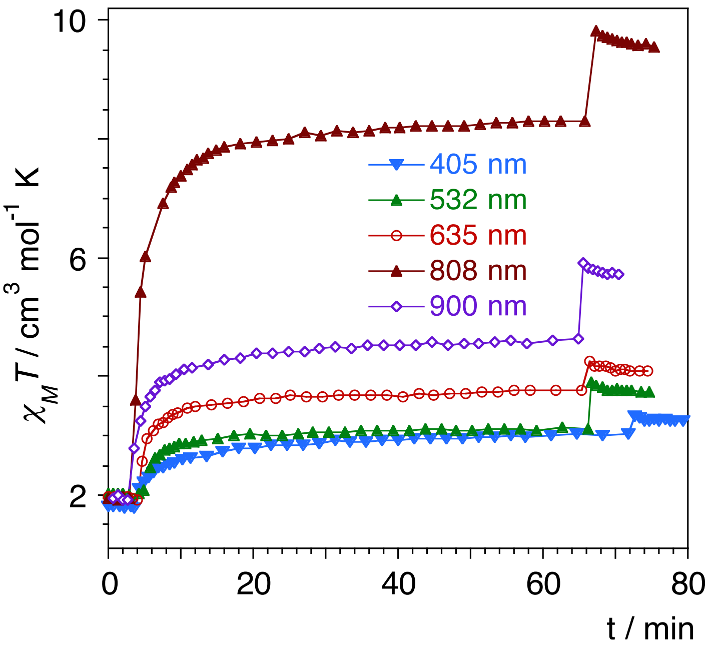

Figure S3. $\chi_M T$ *vs* time curve of **1** under light irradiation at 405, 532, 635, 808, 900 nm at the respective power 5, 7, 10, 8 and 6 mW·cm^{-1}.

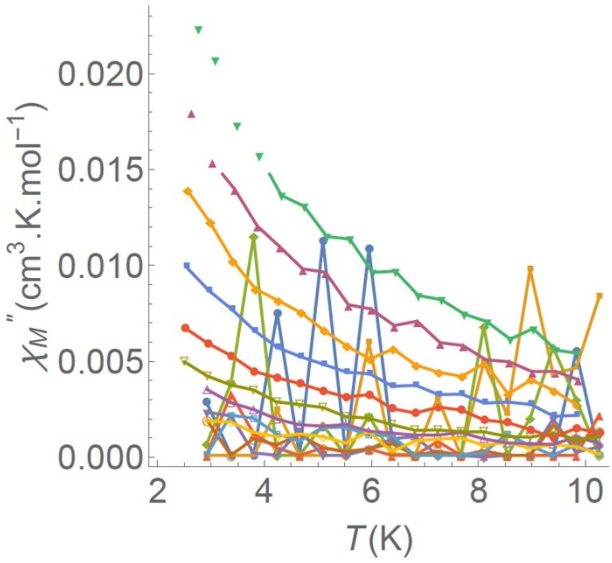

Figure S4a. Out-of-phase component, χ_M'', of the magnetic susceptibility of **1** under a zero dc magnetic field and for frequencies between 30 Hz and 10 kHz.

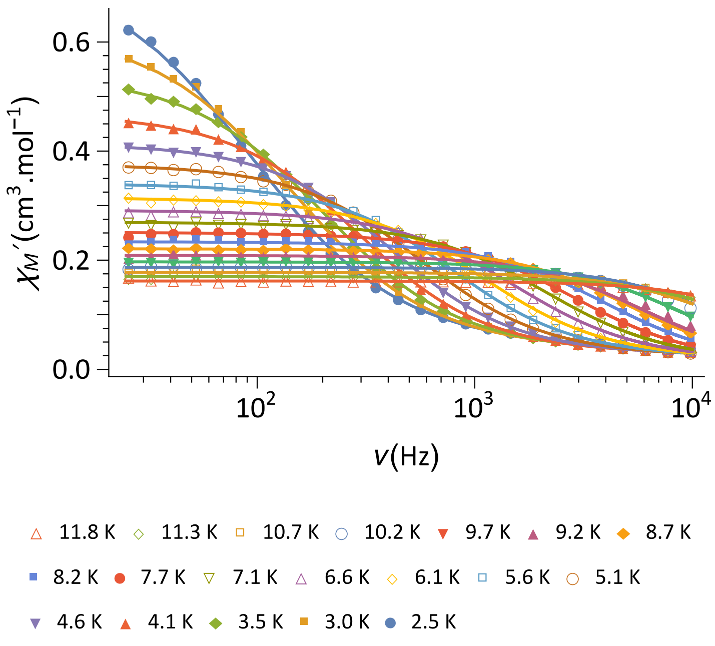

Figure S4b. In-phase component, χ_M', of the magnetic susceptibility of **1** under a 1.8 kOe magnetic field and for frequencies between 30 Hz and 10 kHz. The solid lines are least-squares fitting of the data using generalized Debye model.

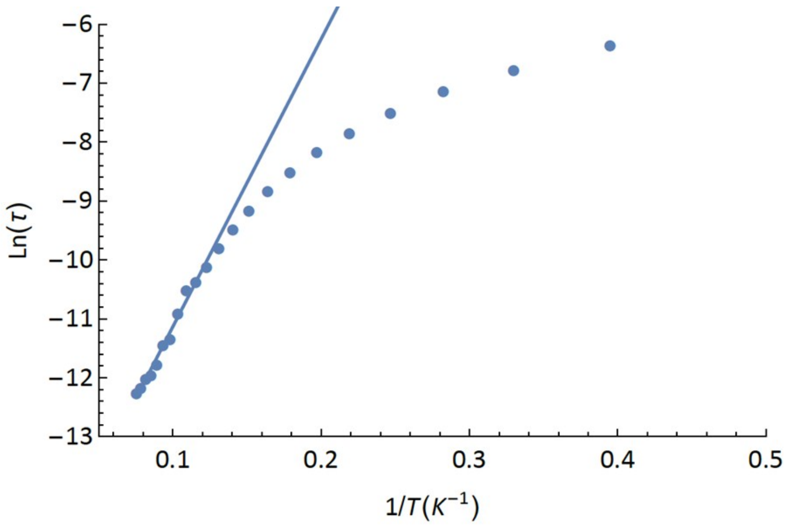

Figure S5. Plot of the natural logarithm of the relaxation time, τ, *vs* the inverse temperature calculated from data at 1.8 kOe. Blue line shows fit of the data for an Arrhenius law between 14 and 7.5 K.

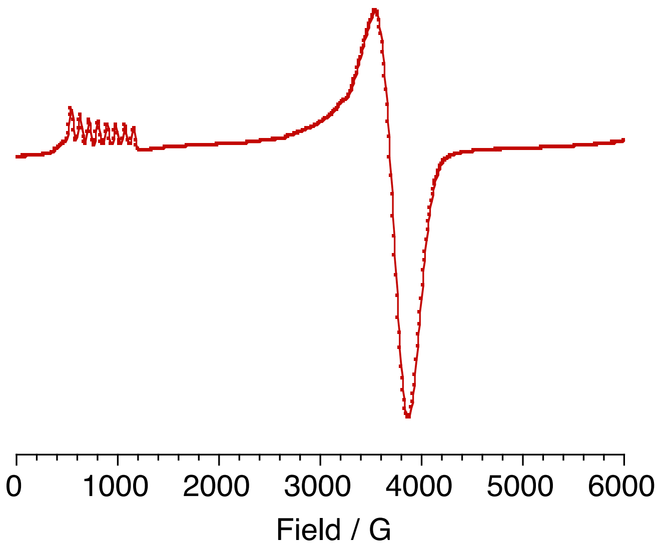

Figure S6. EPR spectrum of **1** in frozen CH_2Cl_2 solution at 4 K at X-band (9.34 GHz).

Section S5 – NMR spectroscopy

^{1}H NMR (400.1 MHz, 298 K, CD_2Cl_2): δ (ppm) = 94.08 (s, 3 H), 37.57 (s, 3 H), 18.46 (s, 6 H), 17.93 (s, 3 H), 15.93 (s, 3 H), 11.41 (s, 3 H), 10.46 (s, 3 H), 9.71 (s, 3+3 H), 8.37 (s, 3 H), 7.96 (s, 3 H), 7.67 (s, 3 H), 1.02 (s, 3 H), -1.47 (s, 6 H), -1.99 (s, 3 H), -2.62 (s, 3 H), -8.43 (s, 6 H), -25.03 (s, 6 H).

^{1}H NMR signal assignment

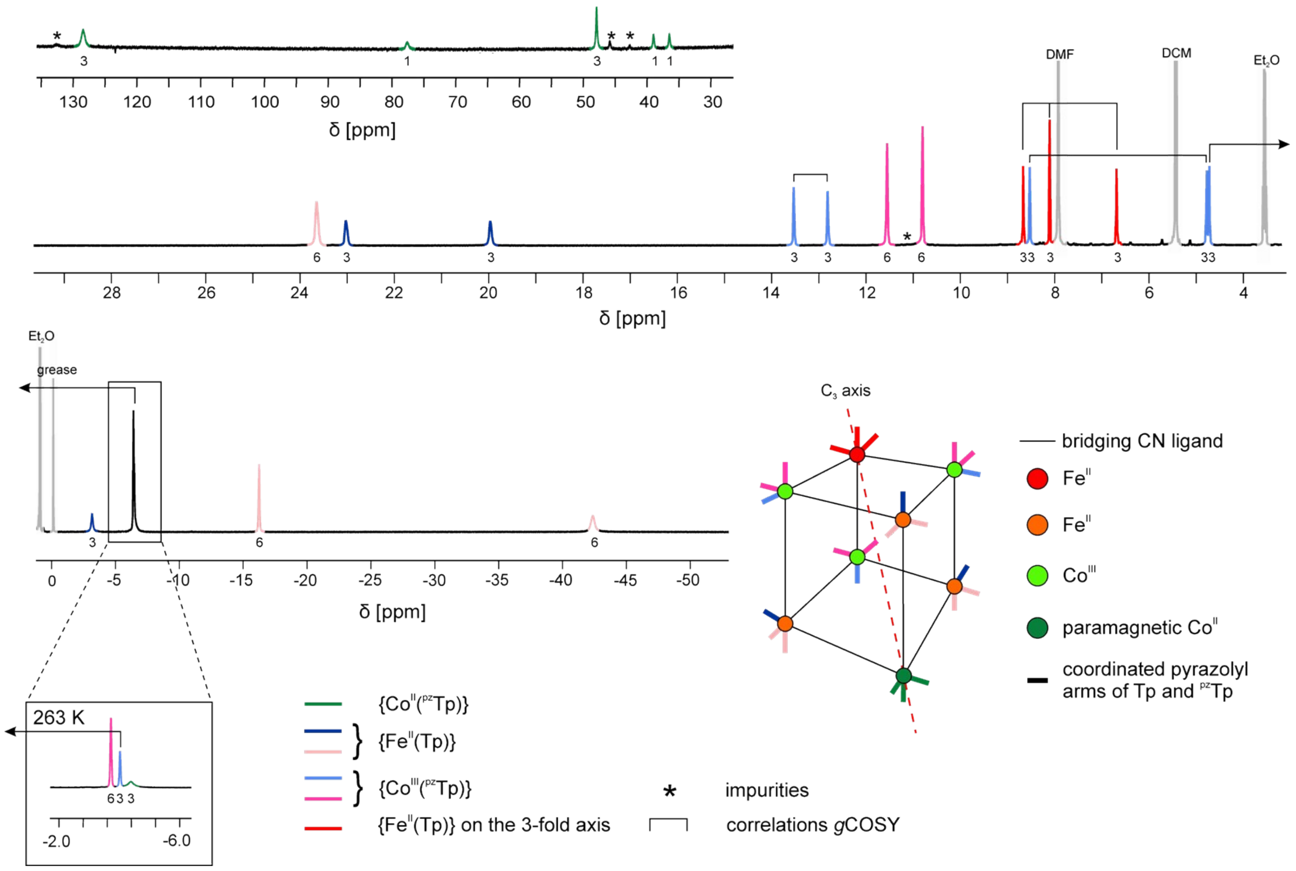

Reminder: Figure 3 of the main article. ^{1}H NMR spectrum at T = 233 K of **1**. Since at this temperature, three signals overlap at δ ≈ -4 ppm, a zoom of the same spectral region at higher temperature (T = 273 K) is depicted in the inset. Partial information about connectivity within pyrazolyl rings when precise attribution is not possible (obtained by *g*COSY) are depicted as brackets.

{Co^{II}(^{pz}Tp)} moiety, paramagnetic green set

Two sets of three ^{1}H signals with relative intensities of 3:3:3 (coordinated pz groups of ^{pz}Tp) and 1:1:1 (uncoordinated pz group of ^{pz}Tp) are expected for the paramagnetic {Co^{II}(Ttp)} moiety. The 1:1:1 set is easily identified thanks to the peak integration. The 3:3:3 set can be identified by searching the signals, which exhibit the largest signal half width among those integrating for 3H. Two of them are located at *ca*. 47 and 128 ppm at 230 K, whereas the third one exhibits a negative chemical and it is located near δ = -5 ppm at 263 K ($\Delta\nu_{1/2}$ ≈ 90 Hz).

{Fe^{II}(Tp)} moiety lying on the 3-fold axis, red set

One set of three ^{1}H signals with relative intensities of 3:3:3 is expected for the {Fe^{II}(Tp)} moiety, which lies on the 3-fold axis. As this moiety is the farthest from the paramagnetic source (connected through three CN bridges to the paramagnetic source), the "more diamagnetic" signals located in the aromatic diamagnetic region of the ^{1}H NMR spectrum at δ = 8.58, 8.03 and 6.60 ppm (at 233 K) can reasonably be ascribed to these protons. It is worth noticing that these signals are almost temperature independent as they shift from 0 to 0.5 ppm over the whole temperature range (115 K). They exhibit so less temperature dependency than the DMF methyl group and the water impurity signals. The three {Fe^{II}(Tp)} signals correlate together in the ^{1}H, ^{1}H *g*COSY spectrum, and even show an unresolved fine structure. Although they show a strong diamagnetic behaviour, they unmistakably belong to **1** as stated by the diffusional NMR studies.

pink set of signals integrating for 6H

The signals highlighted in light pink and fuchsia correspond to the two sets of coordinated pyrazolyl that are equivalent through σ_v-symmetry on the {Fe^{II}(Tp)} and {Co^{III}(^{pz}Tp)} moieties that do not lie on the C_{3v} axis. Their integration corresponds to 6 protons each.

As they do not exhibit crosspeaks in the ^{1}H, ^{1}H *g*COSY spectrum, it is not possible to ascribe with certainty the six signals to the two distinct spin systems; however, reasonable assumptions can be made:

- Three of them (highlighted in light pink) are broader, more strongly shifted and show higher temperature dependency: δ = 23.59, -15.99 and -42.11 ppm (233 K).
- Three others (highlighted in fuchsia), at δ = 11.48, 10.72 ppm (233 K) and -3.72 ppm (263 K) are sharper, tend to show less temperature dependency and are less shifted.

The three signals highlighted in light pink can thus be ascribed to the six pyrazolyl groups facing the cobalt(II) ions and belonging to the {Fe^{II}(Tp)} moieties that are directly connected to the paramagnetic ions through a cyanide bridge. The three signals highlighted in fuchsia are likely due to the six equivalent pyrazolyl groups belonging to the {Co^{III}(^{pz}Tp)} moieties, that are farther away from the paramagnetic centre.

blue set of signals integrating for 3H

The remaining nine signals (in blue), corresponding to three protons each, can be assigned either to the {Fe^{II}(Tp)} moieties or to the {Co^{III}(^{pz}Tp)} moieties, away from the C_{3v} axis.

- ❖ Three signals, highlighted in navy blue, are broader, more strongly shifted and more temperature dependant than the six others: δ = 22.97, 19.90 and -2.92 ppm (233 K).
- ❖ The six remaining signals (highlighted in lighter blue) are sharper, less shifted and less temperature dependant: δ = 13.46, 12.74, 8.45, 4.69, 4.64 ppm (233 K) and -4.02 ppm (263 K). Furthermore, partial correlations could be detected by the ^{1}H, ^{1}H *g*COSY, which is in line with longer nucleus relaxation rates and more diamagnetic behaviours.

Consequently, the navy blue set can be reasonably ascribed to the {Fe^{II}(Tp)} moieties and the lighter blue one to the {Co^{III}(^{pz}Tp)} ones, because they are farther away from the paramagnetic centre. The latter group of signal should again be sorted into coordinated pyrazolyl and uncoordinated pyrazolyl signals. Although ^{1}H *g*COSY provides evidence that the pairs of peaks at δ = 8.45 and 4.69 ppm (233 K), at δ = -4.02 ppm (263 K) and 4.64 ppm (233 K), and δ = 13.46 and 12.74 ppm (233 K) belong to the same spin systems, it is not possible to give their precise attribution.

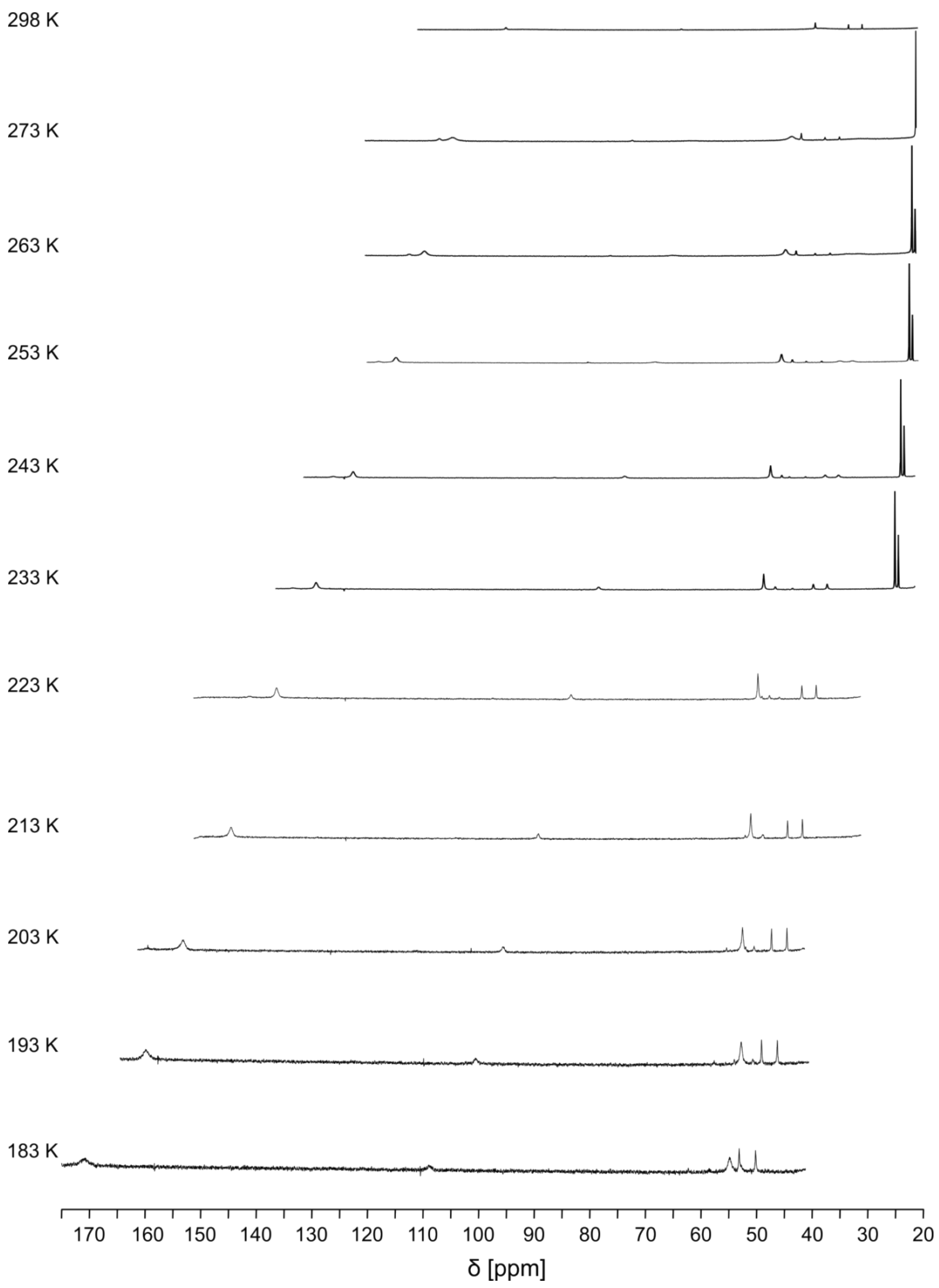

Figure S7. Variable temperature NMR spectra of **1** in CD_2Cl_2 between 298 K and 183 K. Zoom between 175 and 20 ppm.

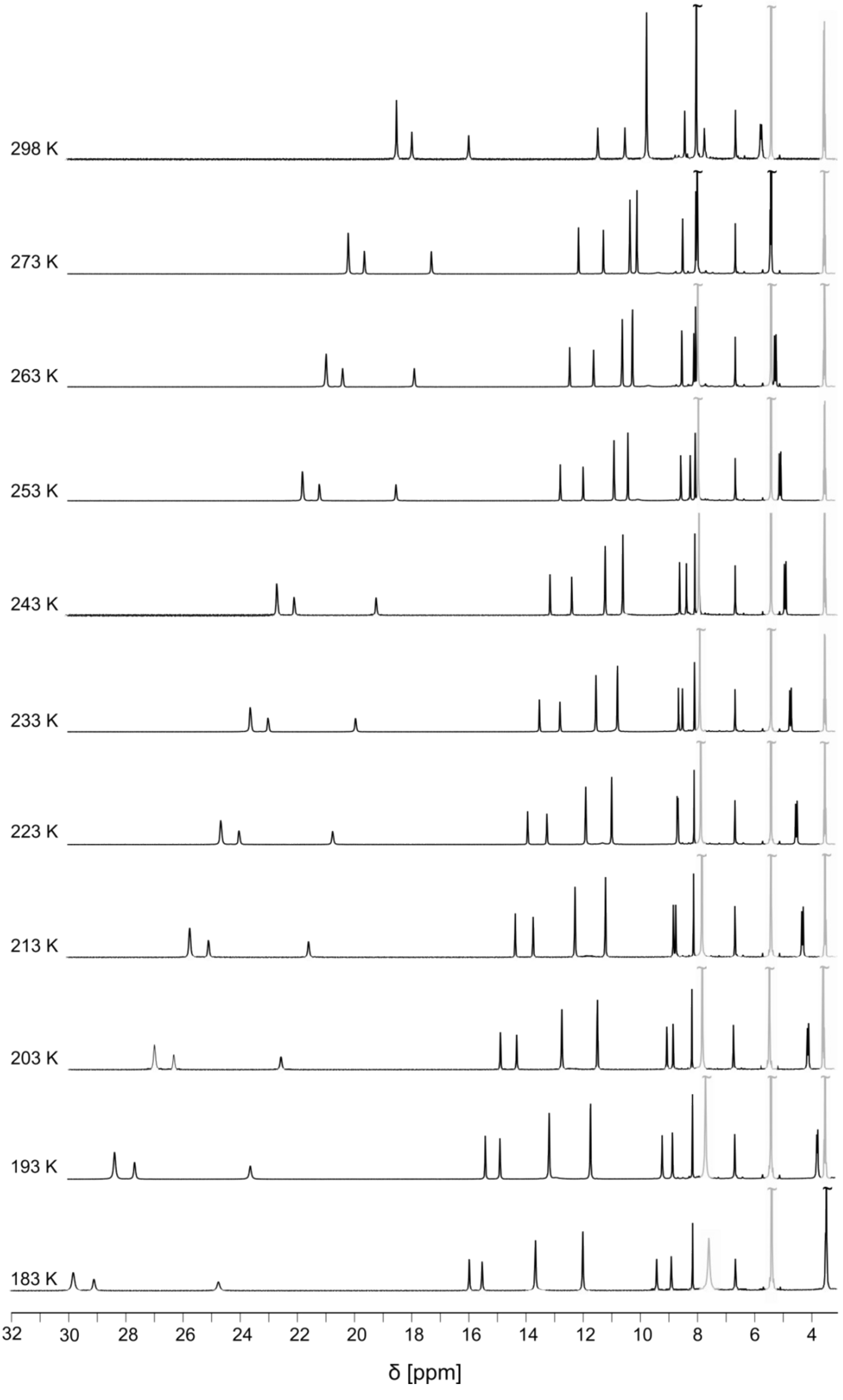

Figure S8. Variable temperature NMR spectra of **1** in CD_2Cl_2 between 298 K and 183 K. Zoom between 3.1 and 30 ppm. The solvent signals are marked in grey when no compound signal lies underneath.

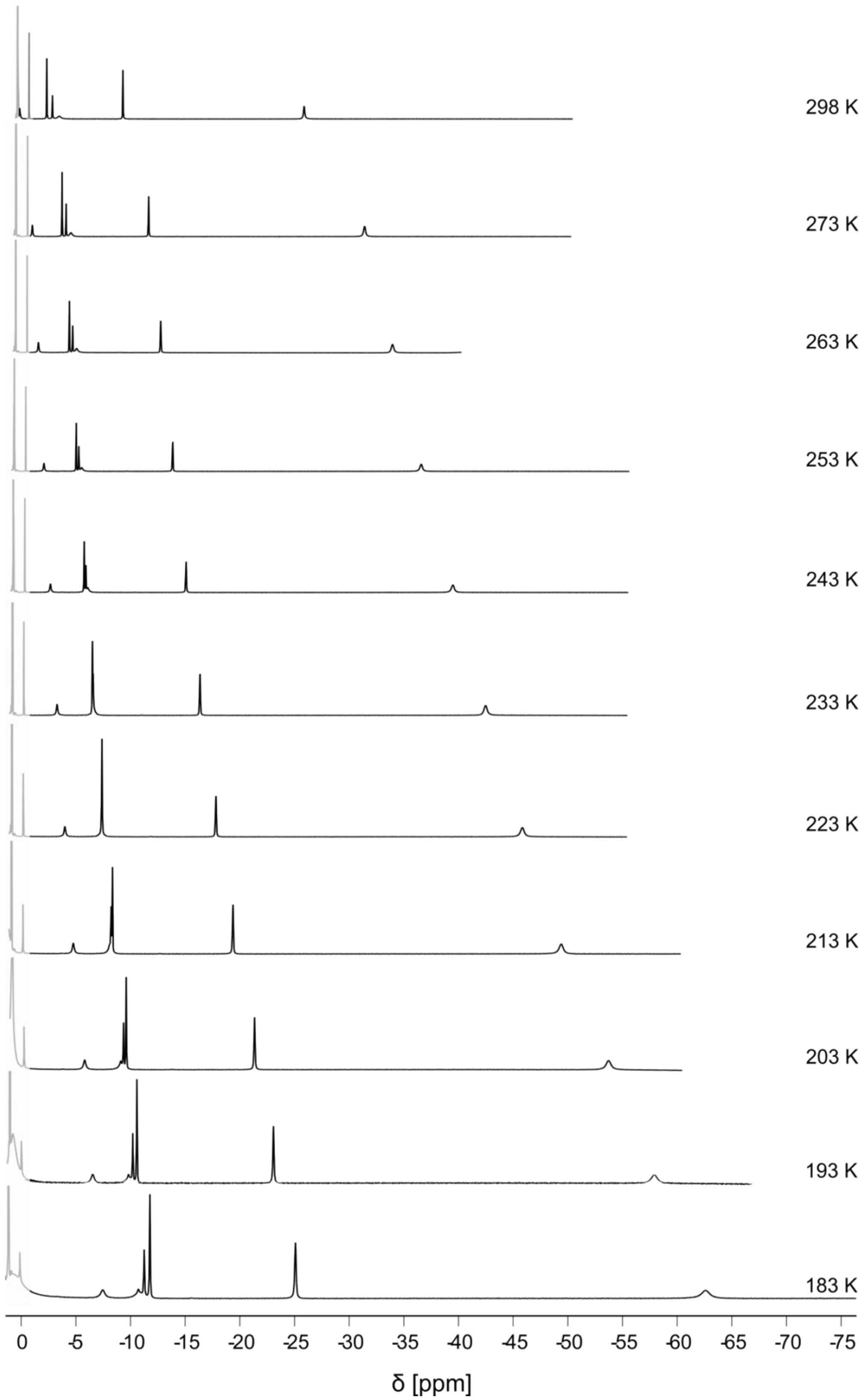

Figure S9. Variable temperature NMR spectra of **1** in CD_2Cl_2 between 298 K and 183 K. Zoom between 1.4 and -76.4 ppm. The solvent signals are marked in grey when no compound signal lies underneath.

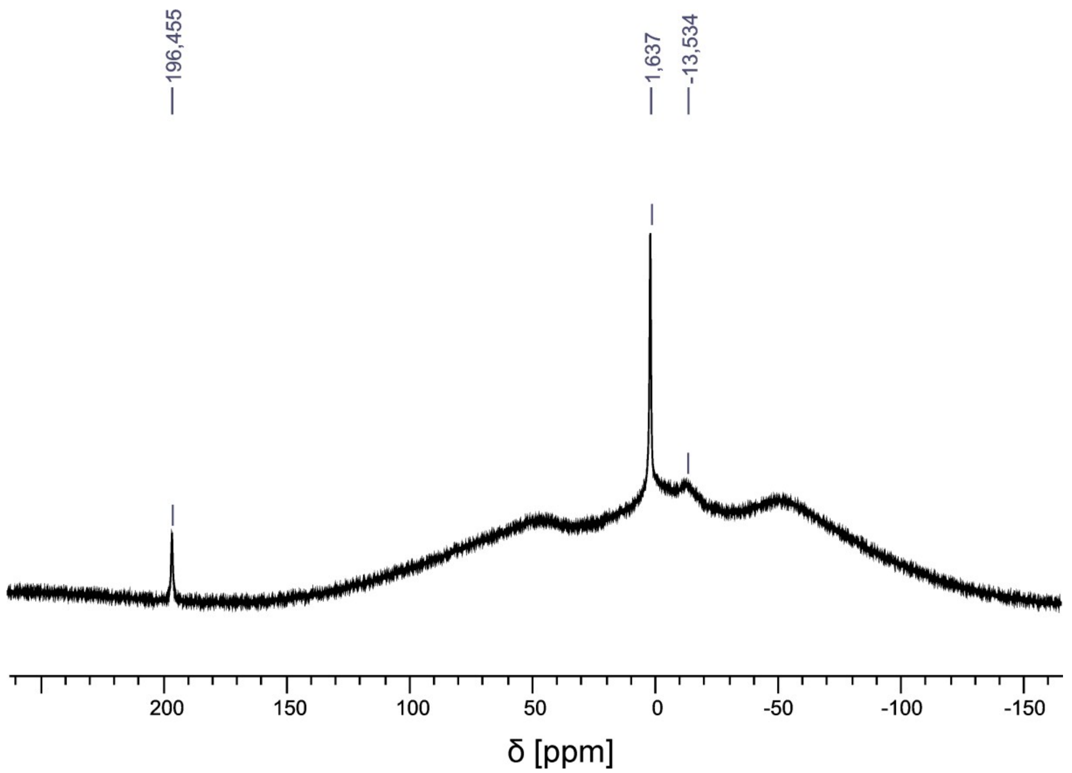

Figure S10. ^{11}B NMR (96.29 MHz) spectrum of **1** in CD_2Cl_2 at 298 K:

δ (ppm) = 196.8 (s, 1B, {Co^{II}(^{pz}Tp)}), 1.85 (s, 3B, {Co^{III}(^{pz}Tp)}, -13.4 (br, s, 4B, {Fe^{II}(Tp)}).

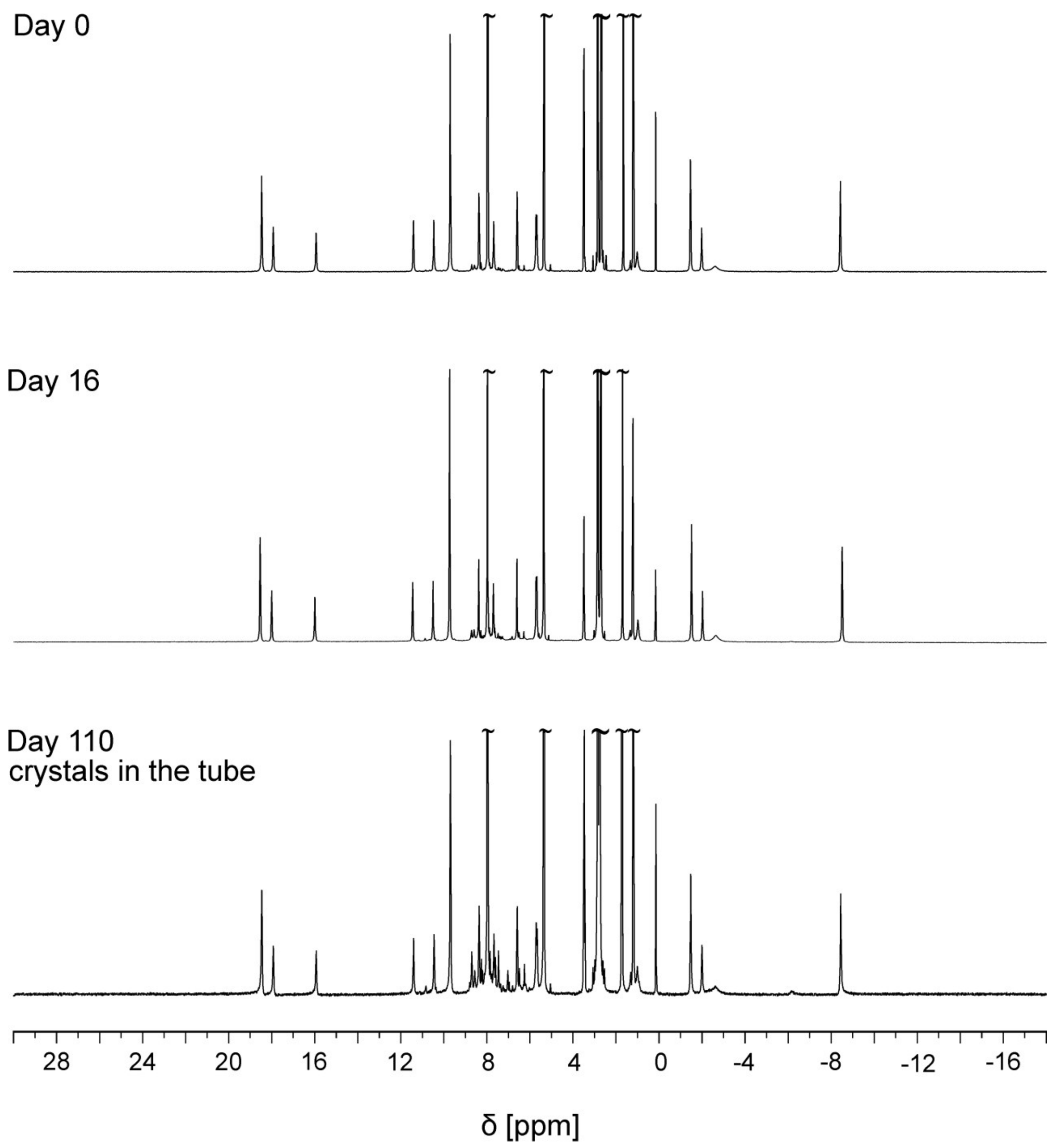

Figure S11. ^1H NMR spectra of **1** in CD_2Cl_2 at room temperature over time.

1 is relatively stable in solution in dichloromethane and can be kept at room temperature, in darkness for several weeks with minimal decomposition.

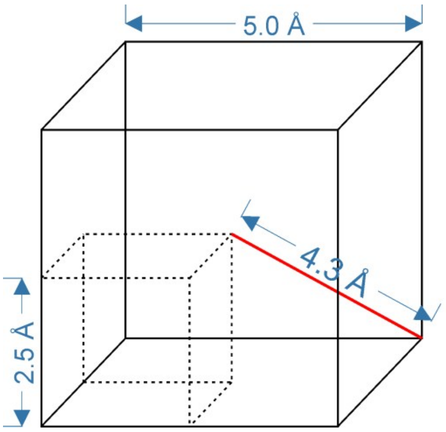

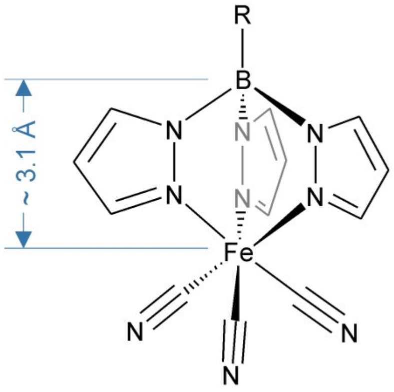

Considering an approximated 5 Å-edged cube, the distance between the centre of the cube and the corners occupied by the metal atoms amounts to 4.3 Å. The typical boron-metal intramolecular distance amounts to 3.1 Å, so that the expected hydrodynamic radius of **1** reaches 7.4 Å. This gives an indication of the size of **1** (the fourth uncoordinated pyrazolyl rings of ^{pz}Tp is not taken into account).

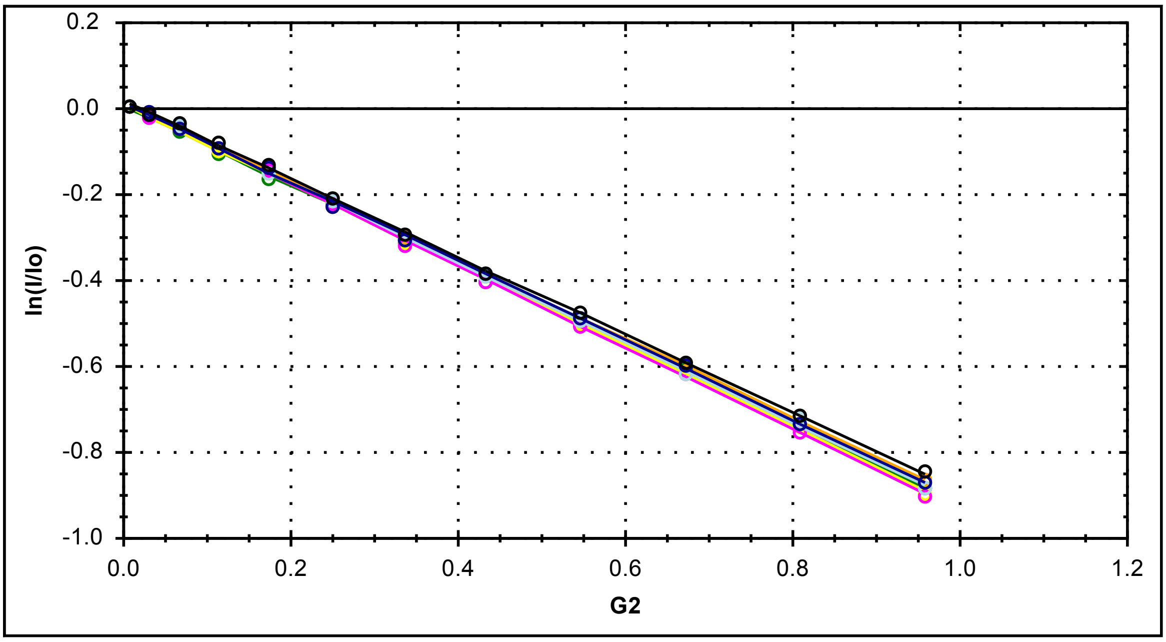

Figure S12. Intensity attenuation curves versus the square of the gradient field for signals at 18.44 (black), 11.41 (marine blue), 10.46 (hell blue), 9.71 (magenta), 8.38 (yellow), 6.61 (green) and -1.42 ppm (orange) at 298 K in CD_2Cl_2 and a diffusion time of Δ = 50 ms. (The hydrodynamic radius is obtained by the Stokes-Einstein equation).

Section S6 – Mass spectrometry

Mass spectrometry ESI-MS *m/z* (%) in CH_2Cl_2: 2779.4 (100) [**1**]$^+$; 2814 (100) [**1**]–Cl^-.

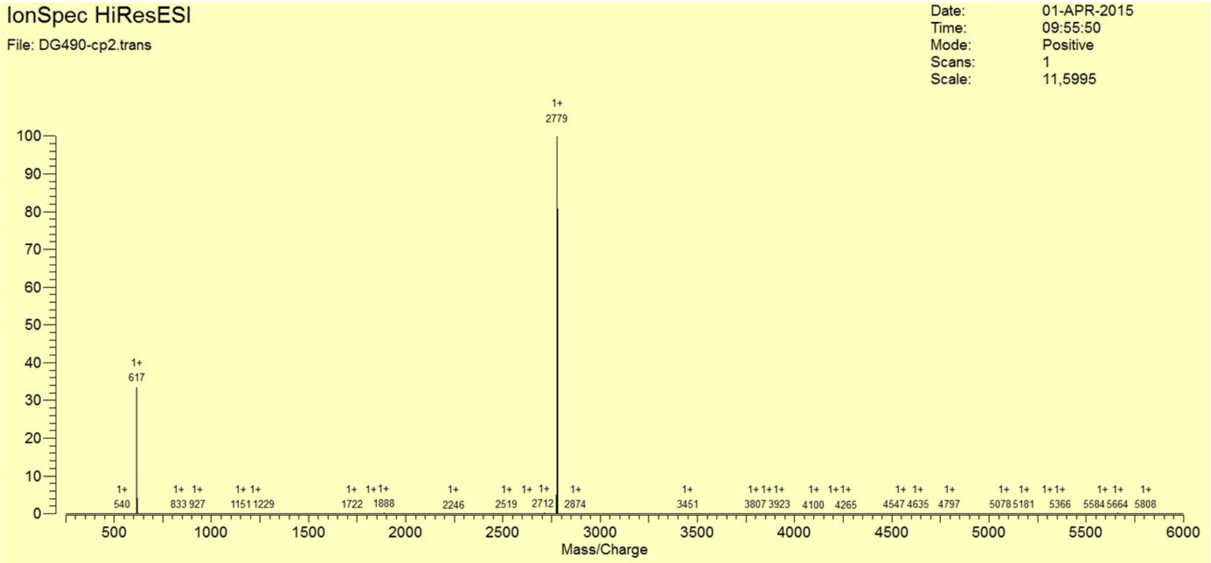

Figure S13a. ESI-MS cationic spectrum of **1** between *m/z* = 250 and 6000 in CH_2Cl_2.

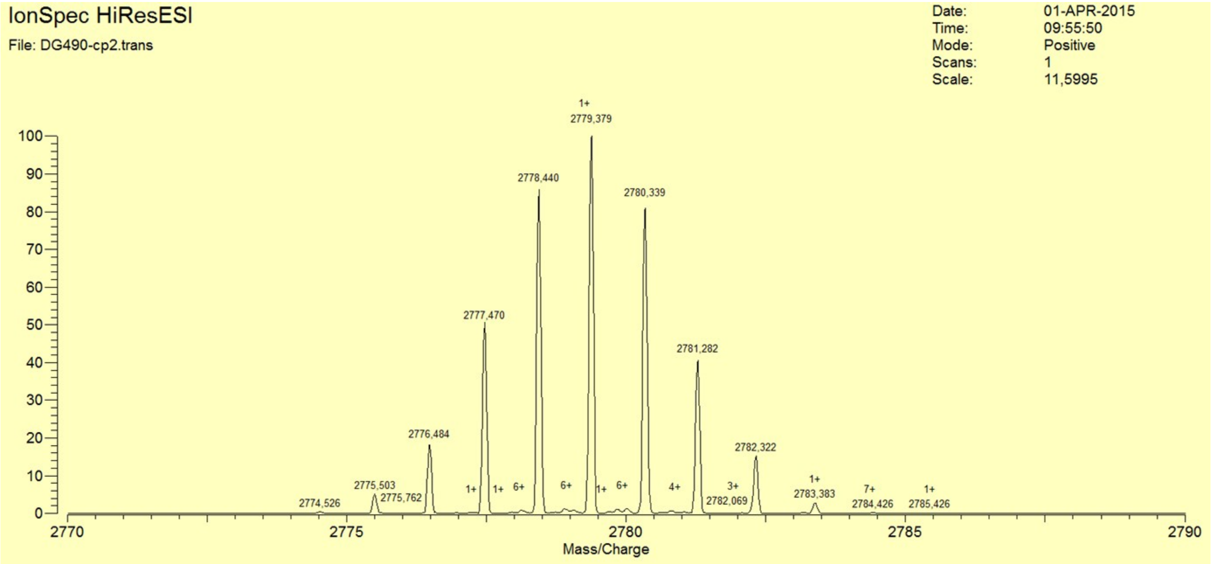

Figure S13b. ESI-MS cationic spectrum, between *m/z* = 2770 and 2790: molecular peak of **1** in CH_2Cl_2.

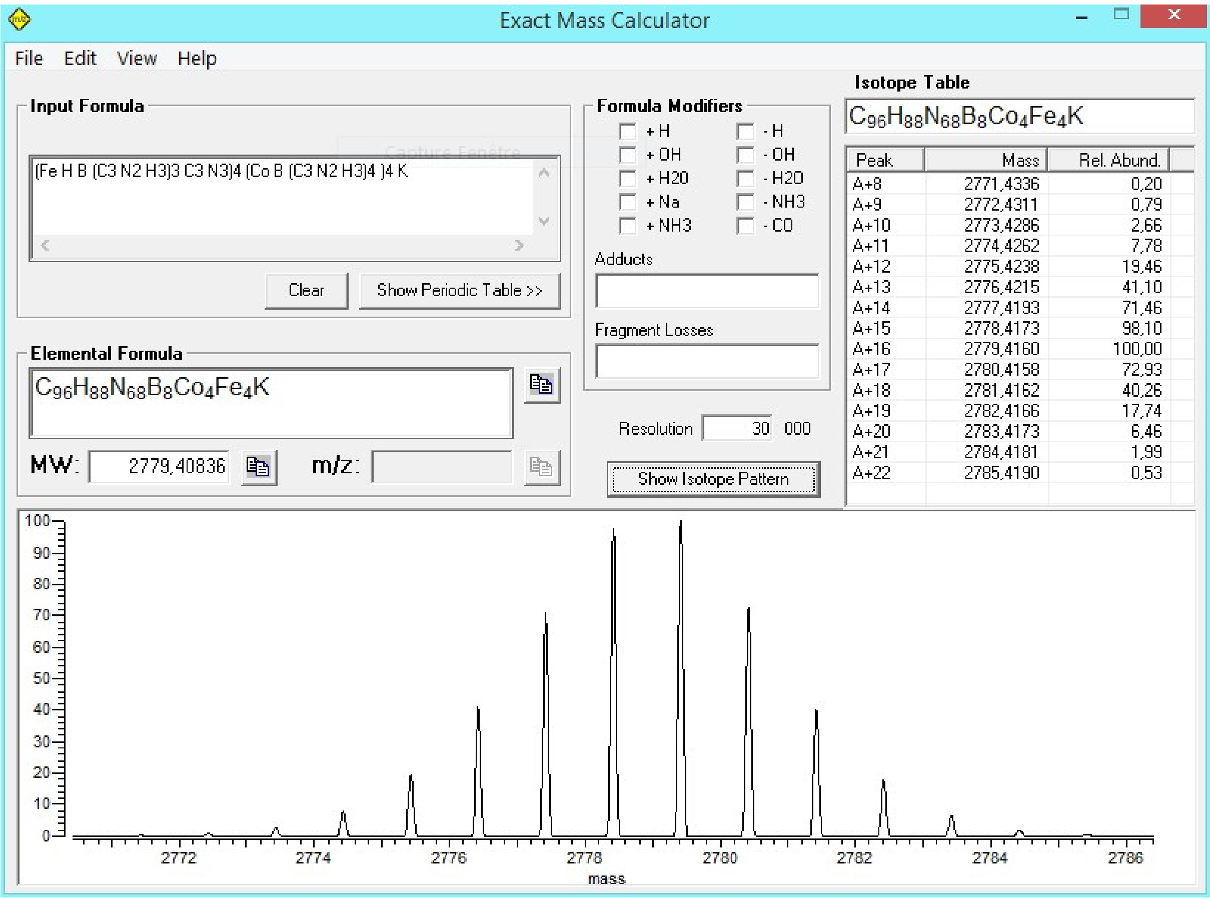

Figure S14. Simulation of the ESI-MS cationic spectrum of **1**.

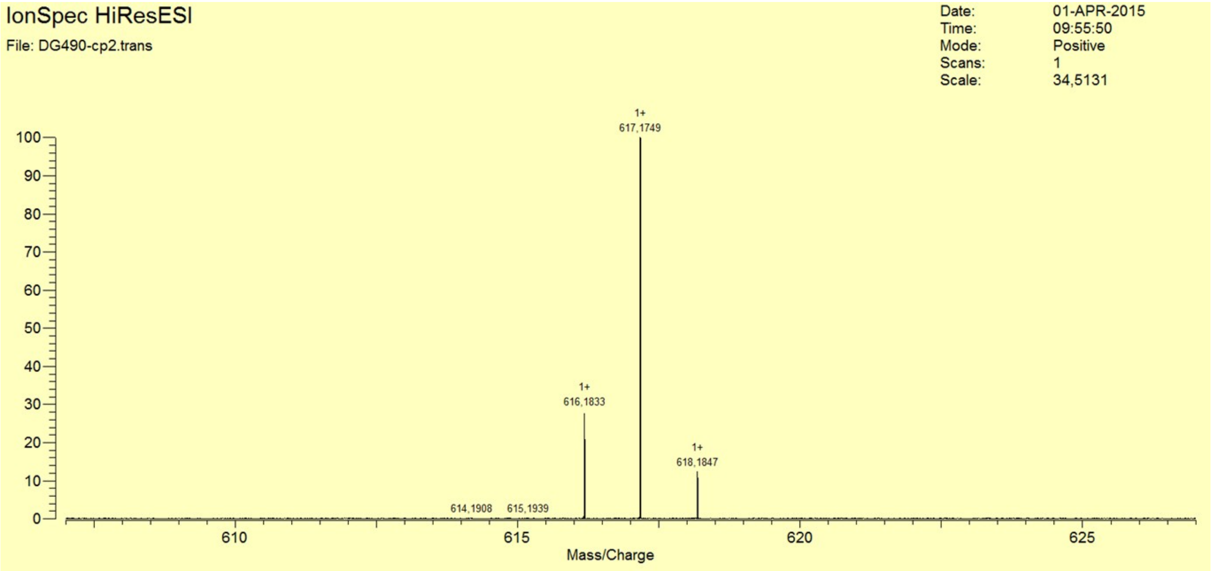

Figure S15. ESI-MS cationic spectrum, between m/z = 607 and 627: cationic impurity of the **1** sample in CH_2Cl_2. It corresponds to $[Co^{III}(^{pz}Tp)_2]^+$, and comes from the last step of the synthesis.

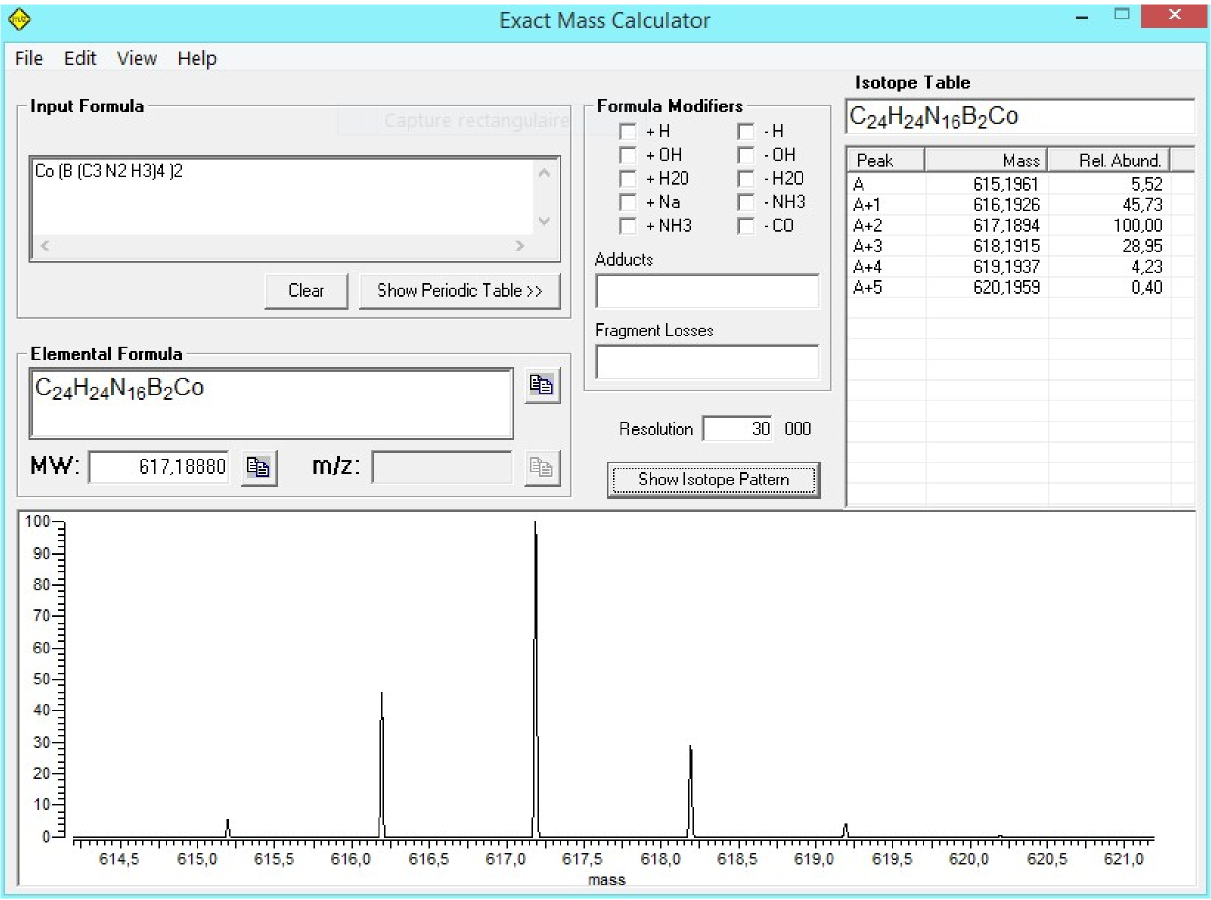

Figure S16. Simulation of the ESI-MS cationic spectrum of $[Co^{III}(^{pz}Tp)_2]^+$.

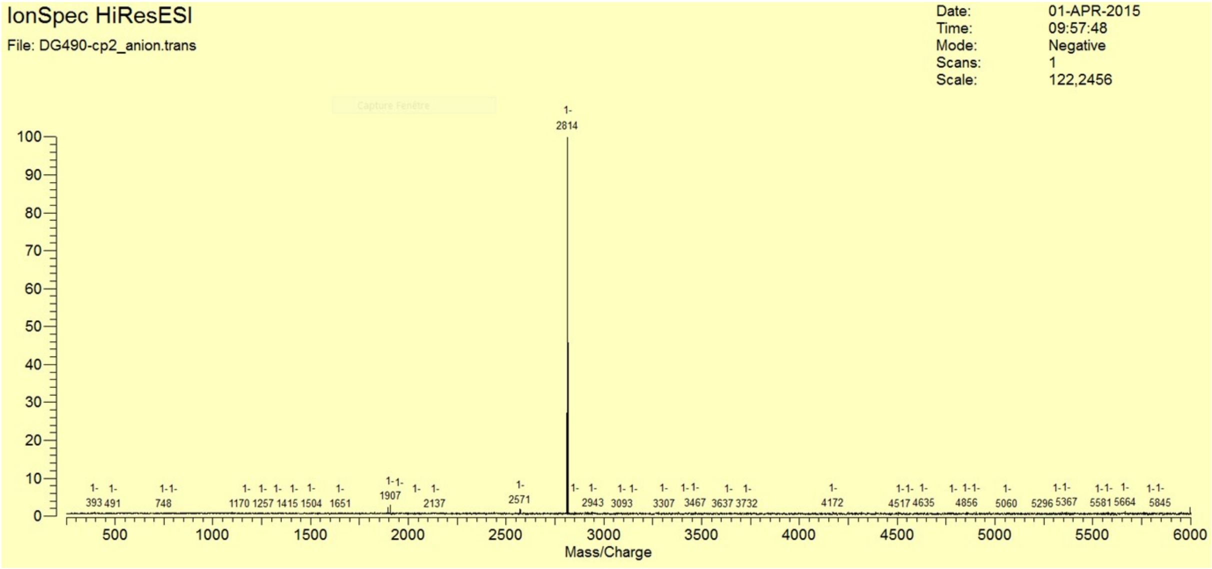

Figure S17. ESI-MS anionic spectrum of **1** between m/z = 250 and 6000 in CH_2Cl_2.

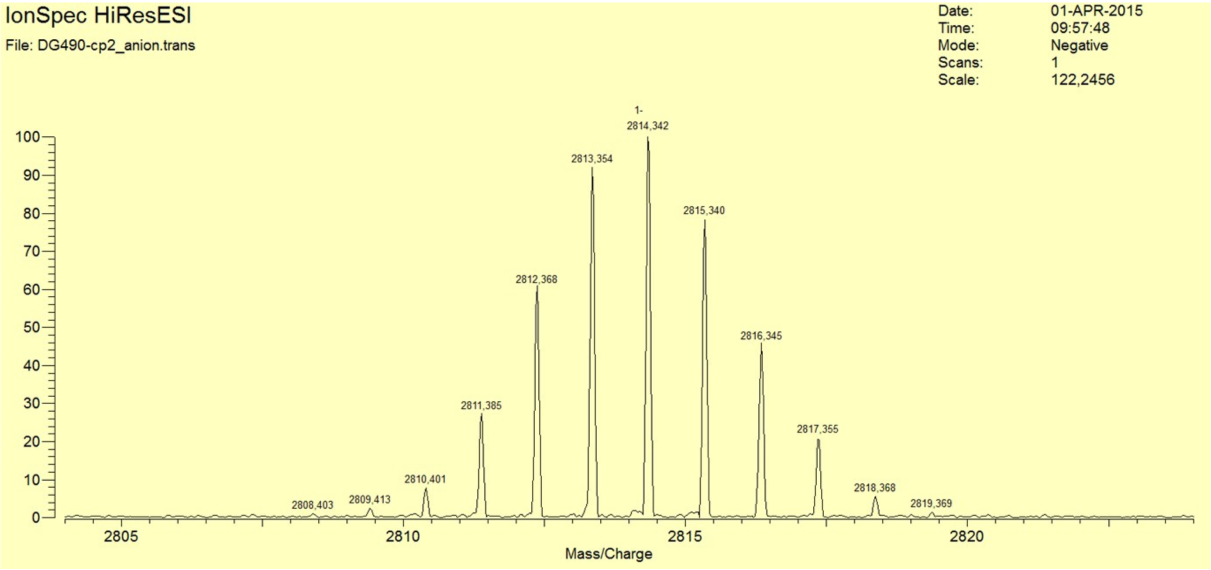

Figure S18. ESI-MS anionic spectrum, between m/z = 2804 and 2824: the signal corresponds to an adduct between **1** and a chloride anion probably coming from CH_2Cl_2.

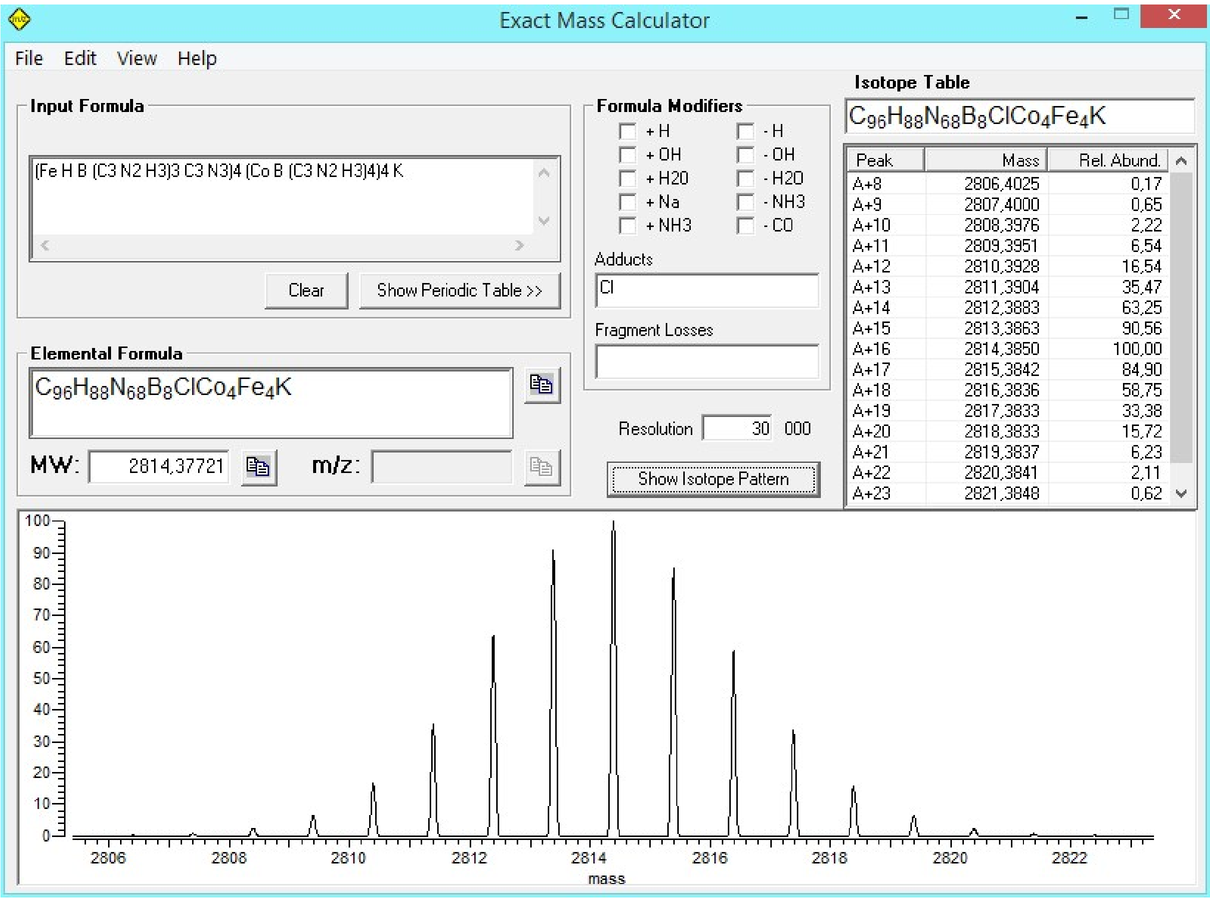

Figure S19. Simulation of the ESI-MS cationic spectrum of [**1**]–Cl$^-$.

Section S7 – Cyclic voltammetry

The oxidation at – 0.02 V is due to the oxidation of the Co^{II} ion. Its corresponding reductive wave is strongly shifted at *ca*. -0.95 mV. Indeed when the potential is not decreased to -1.10 V, the oxidation wave disappears on the second loop (see below). This behaviour is typical of cobalt(II) complexes that exhibit a spin crossover accompanying the redox process: Co^{II}_{HS} <=> Co^{III}_{LS}. It has already be observed in the parent compound $[Co^{II}(^{pz}Tp)_2]$.[S8]

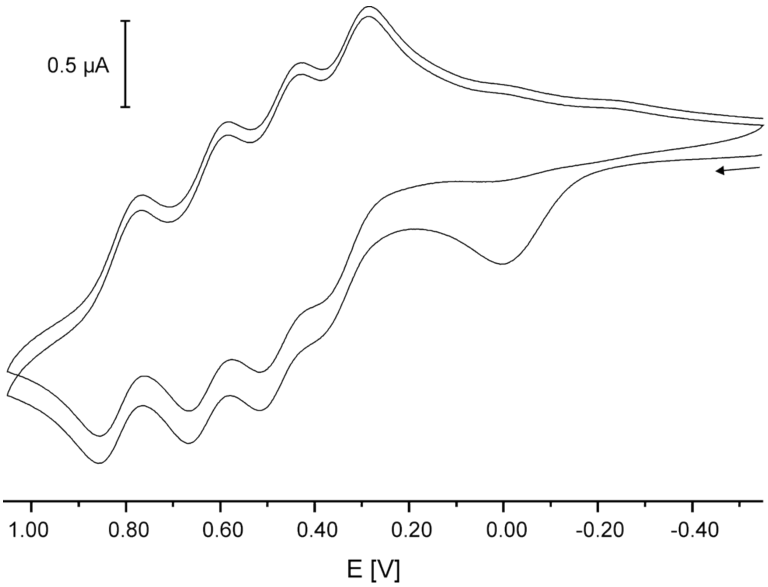

Figure. S20. Cyclic voltammogramm of **1** in CH_2Cl_2, at 250 mV·s^{-1} (*versus* Fc^+/Fc).

The first oxidation process appears to be irreversible when the applied potential is not decreased enough upon reduction, so that the last reduction of the Co^{III}_{LS} at *ca*. -0.95 V is not reached. It is worth noticing that the four oxidation waves of the iron(II/III) ions remain unchanged showing that the complexes is the same.

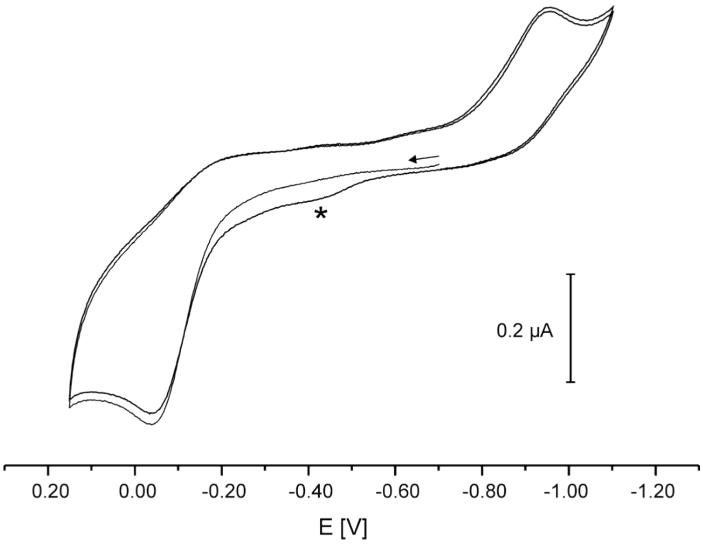

Figure S21. Cyclic voltammogramm of **1** in CH_2Cl_2, at 100 mV·s^{-1} (*versus* Fc^+/Fc).

The cyclovoltammogramm recorded between +0.15 and -1.10 V shows in the present experimental conditions two reproducible waves at + 0.03 V and -0.95 V. They are ascribed to the Co^{II}_{HS} => Co^{III}_{LS} oxidation and Co^{III}_{LS} => Co^{II}_{HS} reduction, respectively. This process appears to be slow because the spin transition, which accompanied the redox process induce a significant structural rearrangement in the cobalt coordination sphere. Even though traces of decomposition due to a concomitant destructive reduction of the cube are detected, both oxidative and reductive processes can be cycled independently from the four quasi-reversible redox processes at higher potential. Indeed the cube molecule decomposes at these low potentials (the signal denoted with a star corresponds to traces of decomposition that are observed when the potential is lowered below *ca*. –1.10 V, see below). The small bump denoted by a star is due to a solvent or electrode impurity.

[S8] D. Chanaka L. De Alwis, Franklin A. Schultz, *Inorg. Chem.*, 2003, **42**, 3616

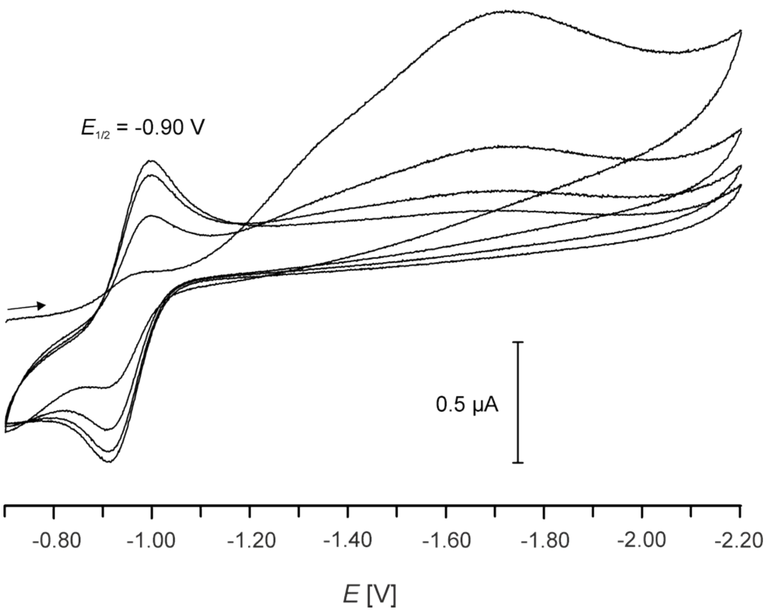

Figure S22. Cyclic voltammogramm of **1** in CH_2Cl_2, at 1000 mV·s^{-1} (*versus* Fc^+/Fc).

The irreversible reductive process gives birth to a new, quasi-reversibly redox-active new species as well as several other redox-active species, inclusive the one responsible for the signal denoted with a star. This quasi-reversible destruction product is ascribed to the $[Fe(Tp)(CN)_3]^{2-/-}$ couple.

Section S8 – UV-visible absorption spectra

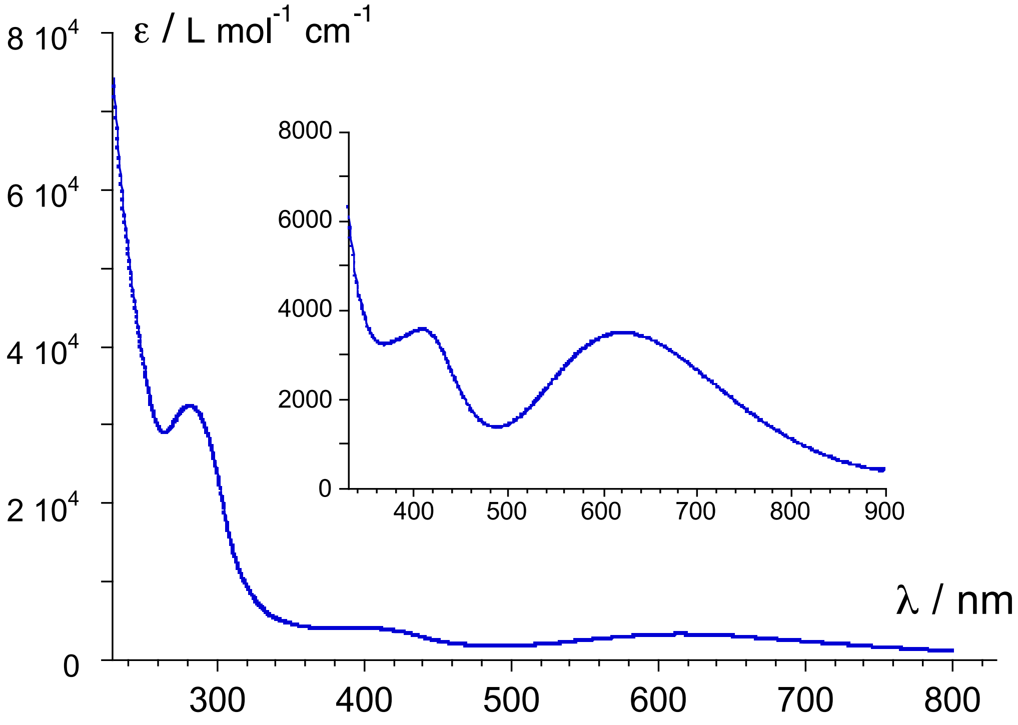

Figure S23. UV-visible absorption spectra of **1** in dichloromethane at room temperature (with C = 14.07 µM and 0.1 mM in inset)

1 features a very strong absorption bands in the near UV and two intense ones in the visible range. The band at λ = 282 nm, (ε_{282} = 32480 L·mol^{-1}·cm^{-1}) is due to intra-ligand transition of coordinated Tp and ^{pz}Tp.
